# Cyprus Sausages’ Bacterial Community Identification Through Metataxonomic Sequencing: Evaluation of the Impact of Different DNA Extraction Protocols on the Sausages’ Microbial Diversity Representation

**DOI:** 10.3389/fmicb.2021.662957

**Published:** 2021-05-17

**Authors:** Eleni Kamilari, Marina Efthymiou, Dimitrios A. Anagnostopoulos, Dimitrios Tsaltas

**Affiliations:** Department of Agricultural Sciences, Biotechnology and Food Science, Cyprus University of Technology, Limassol, Cyprus

**Keywords:** 16S rDNA sequencing, metagenomics, metataxonomic, bacterial diversity, sausages, DNA extraction protocols

## Abstract

Cyprus traditional sausages from the Troodos mountainous region of Pitsilia gained the protected geographical indication (PGI) designation from the European Committee (EU 2020/C 203/06). Still, we lack authentication protocols for the distinction of “Pitsilia” from industrially produced Cyprus sausages. Microbial activity is an essential contributor to traditional sausages’ sensorial characteristics, but whether the microbial patterns might be associated with the area of production is unclear. In the present research, we applied high-throughput sequencing (HTS) to provide a linkage between the area of production and Cyprus sausages’ bacterial diversity. To strengthen our findings, we used three different DNA extraction commercial kits: (i) the DNeasy PowerFood Microbial Kit (QIAGEN); (ii) the NucleoSpin Food Kit (MACHEREY-NAGEL); and (iii) the blackPREP Food DNA I Kit (Analytik Jena), in which we applied three different microbial cell wall lysis modifications. The modifications included heat treatment, bead beating, and enzymatic treatment. Results regarding metagenomic sequencing were evaluated in terms of number of reads, alpha diversity indexes, and taxonomic composition. The efficacy of each method of DNA isolation was assessed quantitatively based on the extracted DNA yield and the obtained copy number of (a) the 16S rRNA gene, (b) the internal transcribed spacer (ITS) region, and (c) three Gram-positive bacteria that belong to the genera *Latilactobacillus* (formerly *Lactobacillus*), *Bacillus*, and *Enterococcus* via absolute quantification using qPCR. Compared with some examined industrial sausages, Pitsilia sausages had significantly higher bacterial alpha diversity (Shannon and Simpson indexes). Principal coordinates analysis separated the total bacterial community composition (beta diversity) of the three Pitsilia sausages from the industrial sausages, with the exception of one industrial sausage produced in Pitsilia, according to the manufacturer. Although the eight sausages shared the abundant bacterial taxa based on 16S rDNA HTS, we observed differences associated with bacterial diversity representation and specific genera. The findings indicate that the microbial communities may be used as an additional tool for identifying of the authenticity of Cypriot sausages.

## Introduction

Cyprus traditional sausages comprise a part of the Cypriot gastronomical culture and are characterized by unique organoleptic characteristics arising from the specific production methods. Their main ingredients include sliced pork meat cured in salt, red wine, and spices. They are formed in a casing made from pork intestine (Ministry of Agriculture, Natural Resources and Environment, Department of Agriculture, 2010). Historically, they were produced in areas of high altitudes such as Pitsilia (Troodos mountain) and Pafos (mountainous regions), whose climate favors the preservation of cured meat products. The main difference in the sausages produced in the two regions is the preservation method applied; sausages in Pafos are preserved by drying, whereas sausages in Pitsilia are preserved, apart from drying, with smoking using wood from indigenous bushes or trees. Pitsilia sausages have been submitted to the European Committee to safeguard their traditional character and achieve to gain the protected geographical indication (PGI) designation (EU 2020/C 203/06). Apart from traditional sausages, industrialized sausages exist in the Cypriot market. Industrially made sausages are cured with cold smoking. Also, nitrate and phosphates are added to provide bacteriostatic and antioxidant properties and to improve the taste and color of the sausages.

Despite their high preference among Cypriot consumers of traditional sausages, the autochthonous microbial communities developed in Cypriot sausages during the process of fermentation and implicated in their unique flavor and texture formation have not been studied yet. The application of high-throughput sequencing (HTS) technology may allow identifying of the microbial diversity of Cypriot sausages. Their capabilities to generate thousands of reading sequences can potentially characterize these complex microbial consortia, including the low-abundance microorganisms. During the last decade, the microbial communities of numerous sausages produced worldwide have been recognized via HTS techniques (Francesca et al., 2013; Hultman et al., 2015; Fontana et al., 2016; Ferrocino et al., 2018).

Before the beginning of fermentation, several microbes dominate the meat microbiota. Lots of them are responsible for spoilage, including several members of the families *Enterobacteriaceae*, *Pseudomonadaceae*, *Actinomycetaceae*, *Bacillaceae*, and *Lactobacillaceae* and lower amounts of other lactic acid bacteria (LAB), Gram-positive, catalase-positive cocci, and yeasts (Francesca et al., 2013; Hultman et al., 2015). During fermentation, LAB produce lactic acid, reducing the pH and, at the same time, inhibiting the population of spoilage microbes. As detected via 16S rDNA sequencing, the most abundant microbes in sausages involved members of the Gram-positive, catalase-positive Staphylococci (GCC++) and lactic acid bacteria, such as members of the families *Lactobacillaceae*, *Streptococcaceae*, and *Leuconostocaceae*. Moreover, spore-forming bacteria, belonging to the family *Bacillaceae*, and spoilage bacteria, including *Pseudomonadaceae*, *Actinomycetaceae*, *Enterobacteriaceae*, and *Moraxellaceae*, are also detected (Benson et al., 2014; Połka et al., 2014; Hultman et al., 2015; Fontana et al., 2016).

A critical step for the efficient analysis of the sausages’ microbiome is to apply a suitable DNA isolation method to achieve a realistic representation of the microbial communities in the sausage matrix. The presence of chemical compounds (polysaccharides, proteins, polyphenols, or lipids) may affect the extracted DNA’s quality or inhibit the downstream PCR analysis (Piskata et al., 2019). Proper sample homogenization is crucial since partial lysis might lead to false bacterial relative representation (Cocolin et al., 2013). The Gram-negative bacterial cell wall is degraded easier than the Gram-positive bacteria (Justé et al., 2008). The commonly used ways for microbial cell wall lysis include a combination of chemical methods with enzymes, such as lysozyme, mutanolysin, or proteinase K regarding bacteria and zymolyase or lyticase regarding fungi. Also, physical disruption with bead beating (40–400 μm for bacteria and 1–3 mm for fungus) is applied. Several commercial kits are now available to isolate bacterial and/or fungal DNA from food products. However, the DNA extraction potential of each kit or each protocol applied varies in quantity and quality of the extracted microbial DNA (Quigley et al., 2012; Henderson et al., 2013; Keisam et al., 2016).

The principal contribution of the present research is to provide a snapshot of Cyprus sausages’ bacterial communities. Our main aim was to distinguish the bacterial diversity of sausages produced in Pitsilia with the industrial sausages using Illumina MiSeq amplicon-based sequencing. Furthermore, we tested the DNA isolation efficacy of three different DNA extraction commercial kits, in which we applied three modifications in the cell lysis step. We evaluated the nine DNA extraction protocols’ efficiency using qPCR assessing the following: (i) the 16S rRNA gene copy number of total bacteria; (ii) the Gram-positive genera *Bacillus*, *Latilactobacillus* (formerly *Lactobacillus*), and *Enterococcus*; and (iii) the internal transcribed spacer (ITS) region copy number. This is the first study performed to characterize the microbial diversity of Cyprus traditional sausages. The obtained findings will enable the characterization of their bacterial diversity and better evaluate their quality and safety for consumers. Notably, the current analysis highlights the bacterial consortia contribution in the definition of Cyprus “Pitsilia” sausages’ typicity.

## Materials and Methods

### Sample Collection

Four sausage samples were collected from three different traditionally cured meat product producers from the area of Pitsilia and one from Pafos (“traditional”) (Table 1). Also, four sausage samples produced from large cured meat industries were collected from the Cypriot market (Table 1) (“industrial”). Two different batches from each source were collected. Samples were transported in ice-cool packs and stored at −20°C until processing.

**TABLE 1:** Information on the sausages used in the present study.

**Sausage group**	**Sample collection area**	**Ingredients**	**Maturation process**
1	Agros, Pitsilia	Pork meat, wine, salt, and spices	They mature in red local wine and then are being smoked.
2	Pelentri, Pitsilia	Pork meat, wine, salt, and spices	
3	Agros, Pitsilia	Pork meat, wine, salt, and spices	
4	Pafos, Cypriot market	Pork meat, wine, salt, and coriander	They mature inside red dry wine and are dried in the sun.
5	Cypriot market (“industrial”)	Pork meat, wine, salt, spices, antioxidants, preservatives, and emulsifiers	They are cured with cold smoking.
6	Cypriot market (“industrial”)	Pork meat, wine, salt, spices, antioxidants, preservatives, and emulsifiers	
7	Cypriot market (“industrial”)	Pork meat, wine, salt, spices, antioxidants, preservatives, and flavor enhancers	
8	Cypriot market (“industrial”)	Pork meat, wine, salt, spices, antioxidants, preservatives, and flavor enhancers	

### Microbiological Culture

We applied culture-dependent methods to isolate and use microbial strains as standards for absolute quantification qPCR experiments. Twenty grams of sausages were homogenized in 180 ml of sterile Buffered Peptone Water (BPW) using Stomacher 400 Circulator (Seward, United Kingdom) at 300 rpm for 5 min. Each sample was serially diluted (1:10 factor). We spread 100 μl from each dilution in four different culture media: (a) nutrient agar (NA) and de Man, Rogosa and Sharpe Agar (MRS), pH = 4 for bacterial isolation, and (b) Rose Bengal Agar (RB) and potato dextrose agar (PDA) for yeasts/fungi isolation. Plates with NA και MRS were incubated at 30°C for 2 days. Plates with MRS were incubated under anaerobic conditions. We incubated plates with RB and PDA at 25°C for 4 days. We purified single colonies by streaking twice. After bacterial and yeast/fungi growth, single colonies were picked, resuspended in 500 μl nutrient broth (NB) and yeast malt extract (YME), respectively, containing 25% glycerol (*v*/*v*), and placed at −80°C.

### Identification of Sausage-Associated Microbes

A 100-μl volume from the pure colonies was added in 10 ml of NB if bacterial, or YME if fungal colonies, and incubated in 30°C for 24 h, or 25°C for 48 h, respectively. Then, 1 ml of each bacterial or yeast culture was placed in a 2-ml tube and centrifuged for 5 min at 12,000 × *g* at 4°C. The pellet was washed for three times in 1 ml sodium chloride (NaCl 0.9%) for purification of potential media residue and diluted in 20 μl lysis buffer (0.25% SDS + 0.05 M NaOH). The dilution was incubated for 10 min at 95°C. Then, 180 μl Tris-HCl (10 Mm Tris-HCl, pH = 8.5) was added, and after centrifugation (5 min 13,000 × *g* in 4°C), the supernatant was placed in a new sterile 2-ml tube.

To identify each bacterial and yeast/fungi isolate, we performed PCR to amplify the 16S rRNA gene and internal transcribed spacer 1 (ITS1) DNA. For PCR experiments, 10 ng/μl of the extracted DNA was used. Primers used for bacterial identification were as follows: 27F (5′-AGAGTTTGGATCMTGGCTCAG-3′) and 1492R (5′-CGGTTACCTTGTTACGACTT-3′), with the following PCR conditions: 95°C for 5 min; 30 cycles of 95°C for 1 min, 62°C for 1 min, and 72°C for 2 min; and 72°C for 10 min. Primers used for yeast/fungal identification were as follows: ITS4 (5′-TCCTCCGCTTATTGATATGC-3′) and ITS5 (5′-GGAAGTAAAAGTGCTAACAAGG-3′) with the following PCR conditions: 95°C for 5 min; 30 cycles of 95°C for 1 min, 55°C for 1 min, and 72°C for 2 min; and 72°C for 10 min. PCR products were sequenced at Macrogen Europe (Netherlands).

### Metagenomic DNA Extraction

A sample of 20 g was suspended in 180 ml of sterile Buffered Peptone Water (BPW) and homogenized using a Stomacher 400 Circulator (Seward, United Kingdom) for 5 min at 300 rpm. Then, 1 ml of the solution was transferred to 2-ml tubes and centrifuged for 1 min at 14,000 rpm. The supernatant was discarded, and the pellet was resuspended in each examined kit lysis buffer. Three commercial food DNA extraction kits were used: (a) blackPREP Food DNA I Kit (Analytik Jena AG) (BP), (b) DNeasy^®^ PowerFood^®^ Microbial Kit (MoBio Laboratories Inc., Carlsbad, CA, United States) (MB) και, and (c) Nucleospin^®^ Food Kit (MACHEREY-NAGEL) (NS), for bacterial and yeast/fungi metagenomic DNA extraction. Additionally, three different cell lysis principles were applied before the DNA extraction process (Table 2):

**TABLE 2:** The three different cell lysis principles that were applied to the three commercial DNA extraction kits.

**Method no**	**Cell lysis principle**	**Lysis agent**	**Commercial kit**
NS_B	Mechanical	Nucleospin^®^ Bead Tubes Type E (3-mm steel και 40–400-μm glass beads)	Nucleospin^®^ Food Kit (MACHEREY-NAGEL)
NS_H	Heating	10 min in 65°C and 10 min in 95°C	Nucleospin^®^ Food Kit (MACHEREY-NAGEL)
NS_E	Enzymatic	25 μl lyticase in 600 μl sorbitol buffer (1.2 M sorbitol, 10 mM CaCl_2_, 0.1 M Tris/HCl pH 7.5, 35 mM β-mercaptoethanol)	Nucleospin^®^ Food Kit (MACHEREY-NAGEL)
BP_B	Mechanical	Nucleospin^®^ Bead Tubes Type E (3-mm steel και 40–400-μm glass beads)	BlackPREP Food DNA I Kit (Analytik Jena AG)
BP_H	Heating	10 min in 65°C and 10 min in 95°C	BlackPREP Food DNA I Kit (Analytik Jena AG)
BP_E	Enzymatic	25 μl lyticase in 600 μl sorbitol buffer (1.2 M sorbitol, 10 mM CaCl_2_, 0.1 M Tris/HCl pH 7.5, 35 mM β-mercaptoethanol)	BlackPREP Food DNA I Kit (Analytik Jena AG)
MB_B	Mechanical	Nucleospin^®^ Bead Tubes Type E (3-mm steel και 40–400-μm glass beads)	DNeasy^®^ PowerFood^®^ Microbial Kit (MoBio Laboratories Inc., Carlsbad, CA, United States)
MB_H	Heating	10 min in 65°C and 10 min in 95°C	DNeasy^®^ PowerFood^®^ Microbial Kit (MoBio Laboratories Inc., Carlsbad, CA, United States)
MB_E	Enzymatic	25 μl lyticase in 600 μl sorbitol buffer (1.2 M sorbitol, 10 mM CaCl_2_, 0.1 M Tris/HCl pH 7.5, 35 mM β-mercaptoethanol)	DNeasy^®^ PowerFood^®^ Microbial Kit (MoBio Laboratories Inc., Carlsbad, CA, United States)

1. Beat beating (B): The pellet-lysis buffer solution was transferred in 2-ml tubes containing 3-mm steel and 40–400 μm glass beads, and beat beating was applied for 10 min at 3,000 rpm.
2. Heating (H): The pellet-lysis buffer solution was incubated for 10 min at 65°C and 10 min at 95°C.
3. Enzymatic lysis (E): The pellet was diluted in 600 μl sorbitol buffer containing 25 μl lyticase and incubated at 30°C for 30 min. The solution was centrifuged for 10 min at 2,000 rpm and the pellet was resuspended in the examined kit lysis buffer.

Then the DNA extraction process was performed according to each kit manufacturer’s instructions. The extracted DNA was stored at −20°C until processing.

### Quantification of Total DNA

**FIGURE 1:**
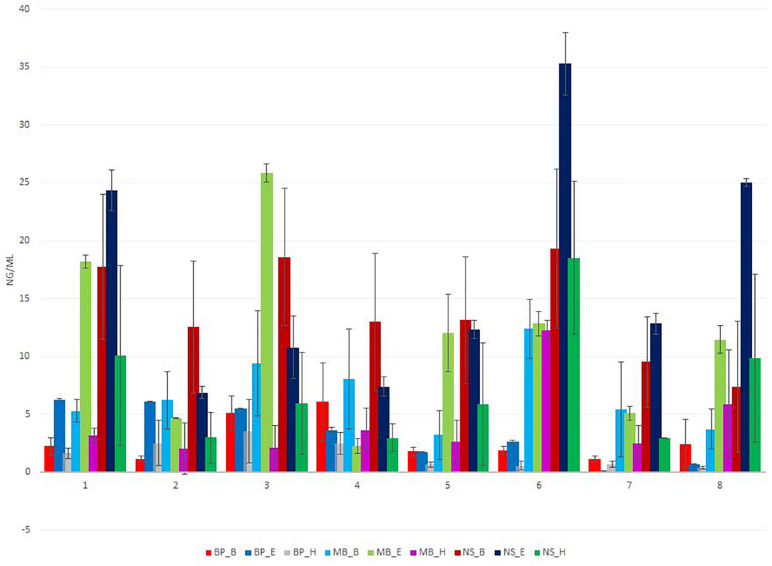
Total extracted DNA yield (ng/μl) for eight different sausage samples isolated using nine different protocols, measured by Qubit. Each of the nine DNA extraction protocols is indicated by a unique color; abbreviations for the samples and the protocols are given in Tables 1, 2, respectively.

**FIGURE 2:**
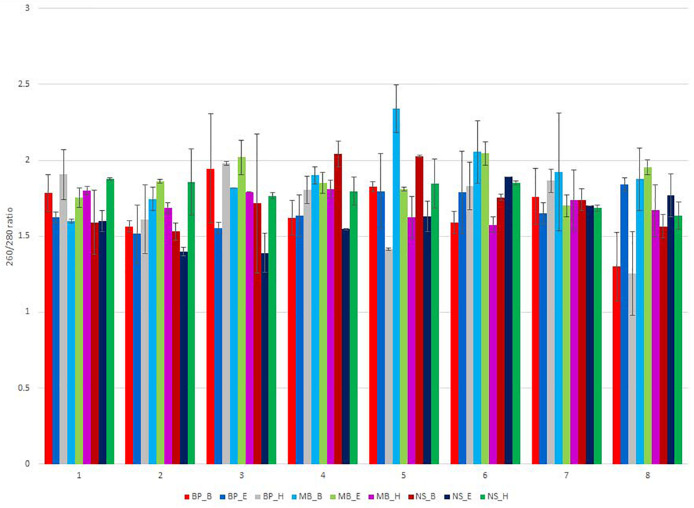
NanoDrop measurement of the 260/280-nm ratio of the isolated DNA, for the eight different sausage samples, extracted using different DNA extraction protocols. Each of the nine DNA extraction protocols is indicated by a unique color; abbreviations for the samples and the protocols are given in Tables 1, 2, respectively.

The total DNA isolated from the sausages was quantified fluorometrically using Qubit dsDNA HS Assay Kit (Invitrogen) with a Qubit 4.0 fluorometer (Invitrogen, Carlsbad, CA, United States) (Figure 1 and Supplementary Tables 3–11). The DNA purity was evaluated by measuring the ratio of absorbance A260/280 nm and A260/230 nm using a spectrophotometer (NanoDrop Thermo Scientific, United States) (Figure 2 and Supplementary Tables 3–11).

### Real-Time qPCR for 16S rRNA Gene and ITS1 Copy Numbers

The qPCR amplification reactions were carried out on the iCycler 5 Real-Time PCR Detection System (Bio-Rad). For every reaction in a 96-well plate, 10 μl of KAPA SYBR FAST Master Mix (2×) Universal (Kapa Biosystems), 2 μl of each primer solution at 5 mM, 5 μl ddH2O, and 1 μl of diluted DNA (1 ng/μl) were used. The selected primers and target genes, as well as the amplification conditions, are shown in Supplementary Table 1. A melt curve was produced for every assay from 60°C to 95°C using the default conditions to confirm the specificity of amplified products. *Latilactobacillus sakei* (*Lat. sakei*) was used as the standard for the total bacterial 16S rRNA gene copy number qPCR experiments. For quantifying the 16S rRNA copy number of the genus *Bacillus*, *Enterococcus*, and *Latilactobacillus*, the isolated DNA of the identified species *Bacillus subtilis*, *Lat. sakei*, and *Enterococcus faecalis*, respectively, were used as standards. *Debaryomyces hansenii* was used as the standard for the total fungal ITS copy number qPCR experiments. For every assay, a calibration curve (*R*^2^ > 0.99) was created to allow the calculation of total bacteria and the three Gram-positive bacteria *Bacillus*, *Latilactobacillus*, and *Enterococcus* 16S rRNA gene copy number, as well as the yeast ITS gene copy number. Standard curves with four 10-fold dilutions were created starting with 1 ng of *B. subtilis*, *Lat. sakei*, and *E. faecalis* 16S rRNA gene as well as 10 ng *D. hansenii* ITS1 region DNA. The calculation of the copy number of *B. subtilis*, *Lat. Sakei*, and *E. faecalis* 16S rRNA gene, as well as *D. hansenii* ITS1 region DNA, was performed based on the following formula: number of copies = (ng × number/mole)/(bp × ng/g × g/mole of bp)^1^. Ct values were generated using the program Bio-Rad iCycler 5 Manager, with default threshold settings. Assay efficiencies were in the range of 0.983–0.999.

### Data Analysis

Statistical differences in the absolute copy number of the 16S rRNA gene and ITS1 DNA region were analyzed using one-way ANOVA, followed by the Tukey–Kramer *post hoc* test and least significant difference (LSD) using SPSS (SPSS Inc., Chicago, IL, United States) (*p* ≤ 0.05).

### Barcoded Illumina MiSeq Amplicon Sequencing of Bacterial 16S rRNA Gene

Using the paired-end approach, 16S rRNA bacterial gene amplification was performed based on the protocol provided by Illumina^2^, as described by Kamilari et al. (2020a). The sequencing run was performed with a MiSeq 600-cycle Reagent Kit v3 (Illumina, United States) (5% PhiX) on a MiSeq Illumina sequencing platform.

### Microbiome and Statistical Analysis

FASTQ sequence analysis, de-multiplexing, alpha diversity metrics (Shannon, Simpson, and Chao1), and beta diversity index (Bray–Curtis dissimilarity) were performed using QIIME 2 version 2020.2 (Bolyen et al., 2019), as previously described (Kamilari et al., 2020b). The distance and compositional dissimilarity matrices were determined through Bray–Curtis dissimilarity distances to visualize the clustering of the bacterial composition for beta diversity analyses. Principle coordinate analysis (PCoA) was estimated using q2-diversity after 99 samples were rarefied (subsampled without replacement) with 25 sampling depths. Differences in bacterial community composition between sausage samples were evaluated using non-parametric permutational analysis of variance (PERMANOVA) (Anderson, 2008). For the clustering and assigning the taxonomy to the 16S rDNA sequences, the 99% cut-off GreenGene database (McDonald et al., 2012) was applied for training the feature classifier. Biomarker discovery analysis was performed using the LEfSe tool (Segata et al., 2011). Linear discriminant analysis (LDA) scores greater than 2.0 were considered significant.

All raw sequence data in read-pairs format were deposited to the National Center for Biotechnology Information (NCBI) in Sequence Read Archive (SRA) under BioProject PRJNA679907.

## Results

### Quantity and Quality of the Extracted DNA From Sausage Assessment

We tested three commercial food DNA extraction kits: (a) Nucleospin^®^ Food Kit (MACHEREY-NAGEL), (b) blackPREP Food DNA I Kit (Analytik Jena AG) και, and (c) DNeasy^®^ PowerFood^®^ Microbial Kit (MoBio Laboratories Inc., Carlsbad, CA, United States) for their effectiveness in isolating DNA that is suitable for qPCR amplification and 16S rDNA metataxonomic amplicon-based sequencing after applying three modifications in the cell lysis step: (a) beat beating, (b) heating, and (c) enzymatic lysis.

The amount of DNA varied based on the extraction protocol (Figure 1 and Supplementary Tables 3–11). The overall DNA yield was in a range of 0.5 to 37 ng/μl of extracted DNA. An amount lower than 5 ng/μl is not sufficient for conducting the first PCR reaction of 16S rDNA metagenomic sequencing. All protocols but blackPREP Food with the modifications heat, enzymatic, and beat beating treatment provided the necessary DNA concentration for 16S rDNA sequencing. The most efficient protocol for the isolation of DNA in most samples was Nucleospin^®^ Food with the modification of an additional bead-beating step during sample homogenization. The least efficient protocols were the blackPREP Food with the heating and bead-beating step modifications.

Sausages contain several contaminants, including protein, salts, and polysaccharides, and the NanoDrop absorbance profile (260/280 nm and 230/260 nm ratio) is suitable for the detection of these contaminants that may interfere and inhibit DNA amplification and sequencing. In some samples, the 260/280 nm ratio of the extracted DNA was out of the limits of 1.8–2.0, indicating the presence of contamination (Figure 2 and Supplementary Tables 3–11). None of the applied protocols was sufficient to eliminate contaminants that absorb at 230 nm, as shown by the ratio 230/260 nm since the extracted DNA was out of the limits of 2.0–2.2 (Supplementary Tables 3–11).

## Real-Time PCR Reaction for Microbial DNA Measurement

### Evaluation of the Efficiency of the Protocols

**FIGURE 3:**
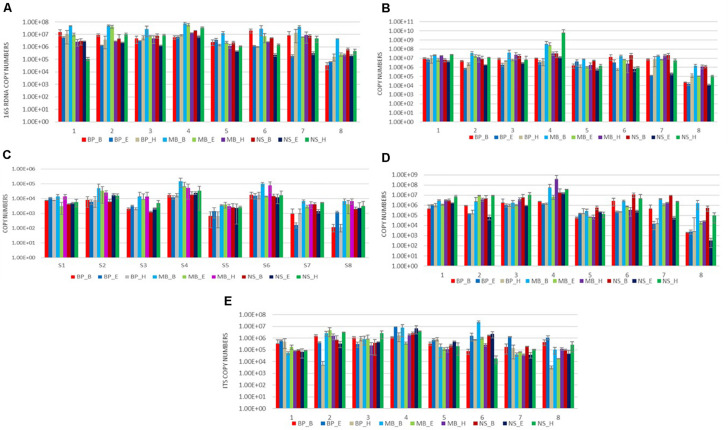
Results of amplification by qPCR of DNA extracted from eight sausages using nine DNA isolation protocols, using five specific primers targeting the following: **(A)** the 16S rRNA gene of total bacteria; **(B)** the 16S rRNA gene of *Lactobacillus*; **(C)** the 16S rRNA gene of *Enterococcus*; **(D)** the 16S rRNA gene of *Bacillus*; and **(E)** the ITS region of fungus. Each of the nine DNA extraction protocols is indicated by a unique color; abbreviations for the samples and the protocols are given in Tables 1, 2, respectively.

We evaluated the nine protocols of DNA isolation for their efficiency to isolate the following: (a) absolute copy number of the 16S rRNA gene; (b) 16S rRNA gene copy number of the Gram-positive bacteria *Latilactobacillus*, *Bacillus*, and *Enterococcus*; and (c) copy number of the ITS loci, in real-time PCR reaction via one-way ANOVA. As can be seen from Figures 3A–C, as well as Supplementary Tables 2–10, a significantly higher average copy number of total bacteria *Latilactobacillus* and *Enterococcus* was detected in the extracts obtained by the MB_B protocol (*P* < 0.05) in most analyzed sausage samples. Regarding *Bacillus*, the extracts obtained by the protocol NS_H and NS_B had significantly higher average copy number compared to the other protocols for most samples (*P* ≤ 0.05) (Figure 3D). Finally, the DNA isolation process with BP_E provided a significantly elevated average copy number of the ITS region (*P* ≤ 0.05) in most sausages’ extracts, in addition to the protocols MB_E, NS_B, BP_H, and MB_B, as can be seen from Figure 3E, as well as Supplementary Tables 2–10.

### Estimation of the Absolute Copy Number of the 16S rRNA Gene and the ITS Loci in Sausages

We compared the absolute copy number of the 16S rRNA gene for total bacteria, *Latilactobacillus*, *Bacillus*, and *Enterococcus*, and of the ITS loci, derived from real-time PCR to evaluate the bacterial and fungal content of the eight sausages. The analysis revealed that sausage 4 had a significantly higher average copy number for the 16S rRNA gene, *Latilactobacillus*, *Enterococcus, Bacillus*, and ITS loci from the other sausages for most of the protocols applied (*P* < 0.05) (Figure 3). Apart from sausage sample 4, sausage sample 6 had also significantly elevated average copy number of Enterococcus.

## Abundance and Alpha Diversity of Members of the Bacterial Microbiota

Ninety-nine examined samples were used as input to the Illumina MiSeq to generate the following: 8,099,286 high-quality sequencing reads, with an average of 81,810.97 sequencing reads per sample (range = 17,809–245,811, STD = 4,615,204.92) and total length of 150 bp (Supplementary Table 12). High-quality sequences were grouped into the average number 97.27 operational taxonomic units (OTUs) (range = 34–624 SD = 75.36). After filtering, we excluded samples with sequencing reads of less than 10,000 from the analysis. Results regarding the alpha diversity indexes (Shannon, Simpson, and Chao1 estimators) are shown in Supplementary Table 12.

**FIGURE 4:**
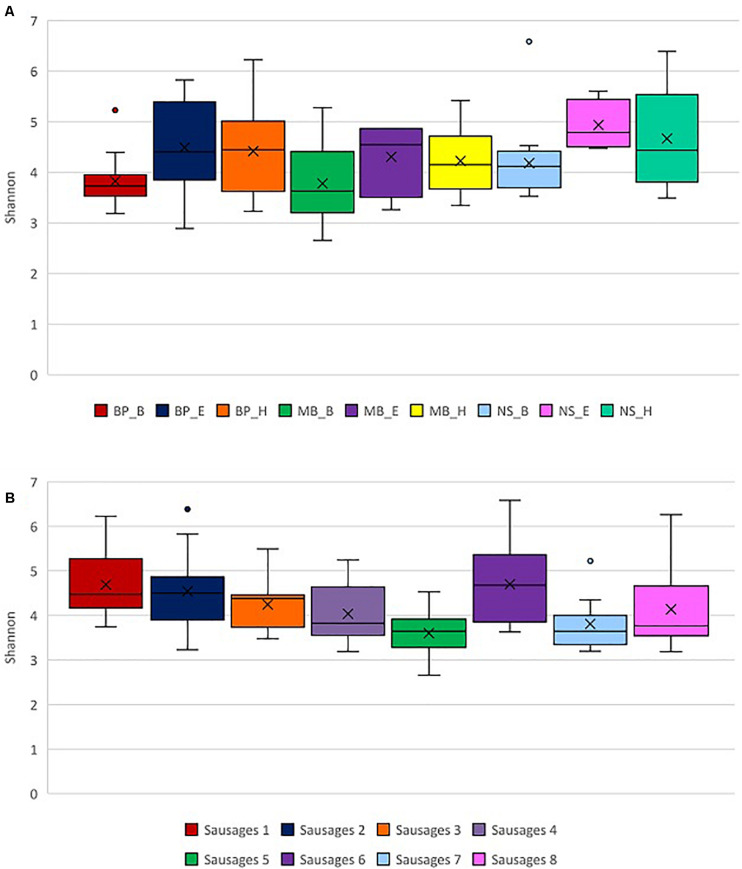
Exploration of alpha diversity based on the Shannon index in sausage samples. **(A)** Comparison of the different sausage samples. **(B)** Comparison of the different protocols of DNA extraction. Statistical analysis was performed using the Kruskal–Wallis test. Each of the eight sausages and of the nine DNA extraction protocols is indicated by a unique color; abbreviations for the samples and the protocols are given in Tables 1, 2, respectively.

Initially, we evaluated the samples of eight different sausage groups’ bacterial alpha diversity based on the Shannon and Simpson indices. Traditional Pitsilia sausages (samples 1, 2, and 3) were characterized by significantly higher bacterial alpha diversity compared to the industrial sausages’ samples 5 (*p* = 0.0001, *p* = 0.006, and *p* = 0.011, respectively) and 7 (*p* = 0.0036, *p* = 0.035, and *p* = 0.043819793, respectively), as indicated by the Kruskal–Wallis test (Figure 4A). Moreover, sausage sample 1 had a significantly higher Shannon index compared to the traditional sausage sample 4 (*p* = 0.019). Finally, sausage sample 6 had a significantly higher Shannon index compared to the sausage samples 4, 5, and 7 (*p* = 0.033, *p* = 0.001, and *p* = 0.012, respectively), based on the Kruskal–Wallis test. The same results were reported for the Simpson index.

Additionally, we compared the impact of the different DNA extraction protocols on alpha diversity as represented by the Shannon and Simpson indices. Both indices indicated significantly higher alpha diversity in samples extracted with the method NS_E compared to the methods BP_B (*p* = 0.003 and *p* = 0.016, respectively), MB_B (*p* = 0.008 and *p* = 0.049, respectively), MB_H (*p* = 0.047 for both indices), and NS_B (*p* = 0.005 and 0.047), based on the Kruskal–Wallis test (Figure 4B and Supplementary Figure 2B). Also, a significantly higher alpha diversity based on the Shannon index was shown in the samples extracted with the method NS_H compared to the methods BP_B (*p* = 0.008) and MB_B (*p* = 0.006), as well as in the samples extracted with the method MB_B compared to the method BP_H (*p* = 0.040), based on the Kruskal–Wallis test.

## Microbial Diversity Distinguishes Pitsilia From the Industrial Sausages

**FIGURE 5:**
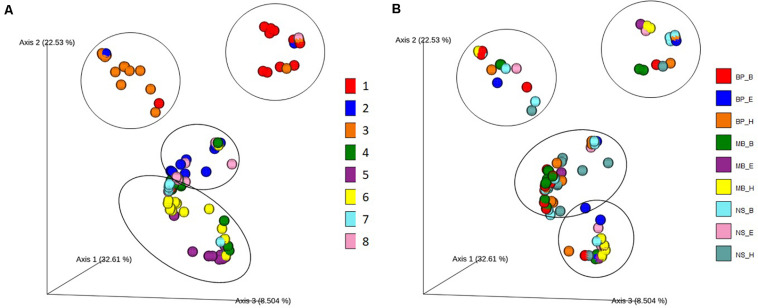
Principal coordinate analysis (PCoA) plot showing the similarities in the bacterial diversity according to the Bray–Curtis distance: **(A)** among the different sausage samples and **(B)** among the different DNA extraction protocols. Each of the eight sausages and of the nine DNA extraction protocols is indicated by a unique color; abbreviations for the samples and the protocols are given in Tables 1, 2, respectively.

To determine the differences in the bacterial diversity among sausage samples and the relative contribution of the different protocols of DNA extraction on bacterial diversity, we employed taxon (OTU)-based measures of beta-diversity. Specifically, a weighted UniFrac distance-based microbiota structure analysis using the Bray–Curtis dissimilarity at the OTU level was performed. Principal coordinate analysis (PCoA) plot of the similarities between the different sausages (Figure 5A) and among the DNA extraction protocols (Figure 5B) was derived based on UniFrac distance. Principal coordinates 1, 2, and 3 explained 32.34%, 22.32%, and 8.41% variance, respectively.

Traditional Pitsilia sausages were distinguished from the other sausages based on the present microbial consortia (Figure 5). An exception was the industrial sausage sample 8, which according to the manufacturer is of “traditional Pitsilia” (although not following the approved PGI norm and not made in the Pitsilia region), that were related to the traditional Pitsilia sausage sample 2. A permutational multivariate analysis of variance (PERMANOVA) test (see Supplementary Table 13) confirmed that the microbial composition is significantly different between Pitsilia sausage 3 samples and sausage 4, 5, 6, 7, and 8 samples (pseudo-*F* = 2.06, *p* = 0.016; pseudo-*F* = 2.30, *p* = 0.003; pseudo-*F* = 2.32, *p* = 0.002; pseudo-*F* = 1.68, *p* = 0.03; pseudo-*F* = 1.53, *p* = 0.04, respectively) and Pitsilia sausage 1 samples and sausage 5 and 6 samples (pseudo-*F* = 1.86, *p* = 0.014; pseudo-*F* = 1.79, *p* = 0.028, respectively). Also, Pitsilia sausage 2 samples indicated a significant difference with sausage 4 samples (pseudo-*F* = 1.98, *p* = 0.025), sausage four samples with sausage 5, 6, and 8 samples (pseudo-*F* = 3.50, *p* = 0.001; pseudo-*F* = 2.51, *p* = 0.011; pseudo-*F* = 1.98, *p* = 0.025, respectively), and sausage 5 samples to sausage 6 and 7 samples (pseudo-*F* = 1.76, *p* = 0.031; pseudo-*F* = 1.74, *p* = 0.028, respectively).

On the other hand, the microbial consortia did not suffice to separate the different analysis protocols into clusters (Figure 5B). Still, the method of DNA isolation affected the sausage clustering, as indicated in Figure 5A. For instance, two extracts from sausage 1 samples (depicted in red color), which were isolated using the BP_H and NS_H protocols, were clustered separately from the other sausage 1 extracts.

## Taxonomic Composition of Bacterial Communities in Sausage Samples, Based on the Different DNA Isolation Protocols

**FIGURE 6:**
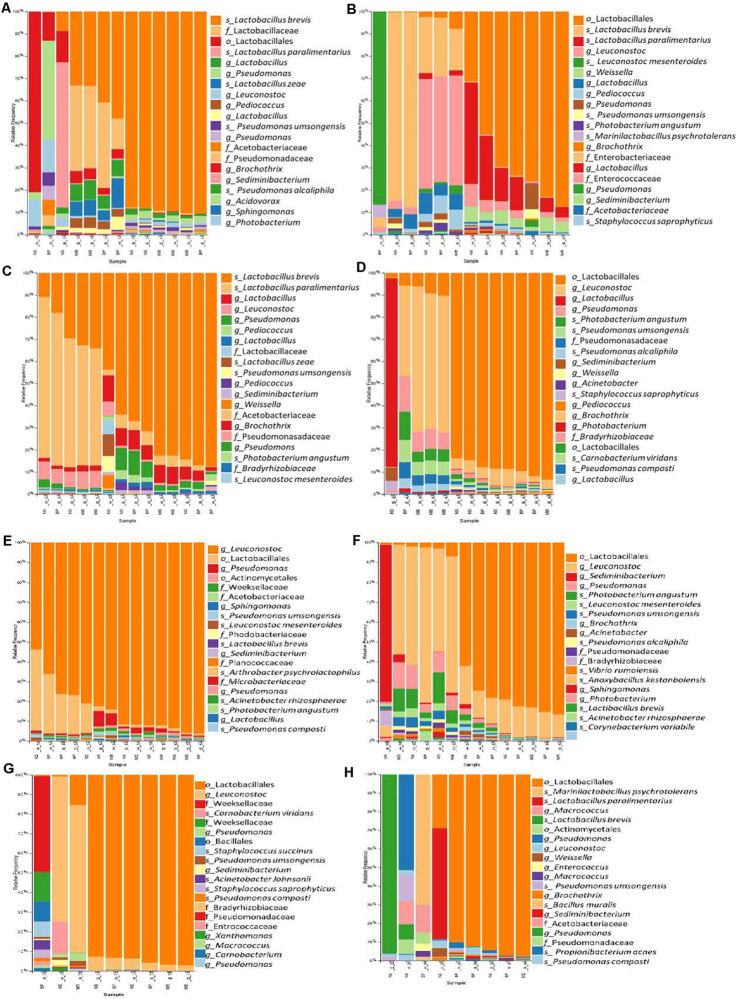
The relative abundance of the 20 most abundant bacteria identified at the species level based on 16S rDNA sequencing for the sausages: 1 **(A)**, 2 **(B)**, 3 **(C)**, 4 **(D)**, 5 **(E)**, 6 **(F)**, 7 **(G)**, and 8 **(H)**. Abbreviations for the samples are given in Table 1.

According to 16S rRNA gene sequencing, Cyprus sausages’ bacterial communities mainly consisted of four bacterial phyla, including mainly Firmicutes and in lower relative abundances Proteobacteria Actinobacteria, and Bacteroidetes. The most commonly detected families belonged to the order Lactobacillales, including *Lactobacillaceae* and *Leuconostocaceae*, with a relative representation of more than 90% in most samples. Another commonly observed family was *Pseudomonadaceae*. The predominant genera included *Latilactobacillus* and *Leuconostoc* and in lower relative abundances the genus *Pseudomonas* (Figure 6 and Supplementary Figure 3). The 16S rDNA sequencing analysis revealed differences in the bacterial diversity among sausage samples. Additionally, the different DNA isolation protocols indicated differences in the relative representation of the same sausage sample’s bacterial communities. Some methods did not suffice to provide taxonomic information beyond the family level (in genus and species level).

Traditional Pitsilia sausage samples 1 and 3 were characterized by an increased relative abundance of the genus *Latilactobacillus*. In contrast, in sausage sample 2, some samples indicated an increased representation of *Leuconostoc* (Figures 6A–C and Supplementary Figures 3A–C). The most representative species were *Levilactobacillus brevis* (*Lev. brevis*) and *Companilactobacillus paralimentarius* (*Com. paralimentarius*, formerly *Lactobacillus brevis*), whereas reads corresponding to the species *Lactobacillus zeae* and *Leuconostoc mesenteroides* (*Leuc. mesenteroides*) were also found. Additional detected LAB included the genera *Pediococcus*, *Weissella*, and *Marinilactobacillus*, which were represented by the species *M. psychrotolerans*, as well as members of the family *Enterococcaceae.* Moreover, the spoilage genus *Pseudomonas*, represented by the species *P. umsongensis* and *P. alcaliphila*, *Acetobacter*, *Brochothrix*, and members of the family *Enterobacteriaceae* were also detected. Additionally, observed contaminants included *Photobacterium*, *Sedimentibacterium*, *Sphingomonas*, *Morganella*, *Chryseobacterium*, *Carnobacterium*, *Bacteroides*, *Staphylococcus*, and *Propionibacterium*.

Similar bacterial communities were detected in the industrial sausages, but in different relative representations (Figures 6D–H and Supplementary Figures 3D–H). Specifically, the industrial sausage sample 5 was characterized by the predominant presence of the genus *Leuconostoc*, in which reads corresponding to the species *Leuc. mesenteroides* were detected. Increased representation of *Leuconostoc* was also found in the industrial sausages’ samples 4, 6, and 7. Also, extracts from sausage samples 5 and 7 showed an increased number of reads that belonged to the family *Weeksellaceae*. Some species were detected in some sausages’ samples; for instance, *Staphylococcus saprophyticus* and *Carnobacterium viridans* were identified only in samples from sausage samples 2, 4, 7, and 8. The species *Acinetobacter rhizosphaerae* was found only in sausage samples 5, 6, and 8; the species *Vibrio rumoiensis* in samples 1, 2, 4, 5, and 6; and the species *Anoxybacillus kestanbolensis* in samples 5, 6, and 8. Finally, the species *Staphylococcus succinus* was only detected in samples 7 and 8.

**FIGURE 7:**
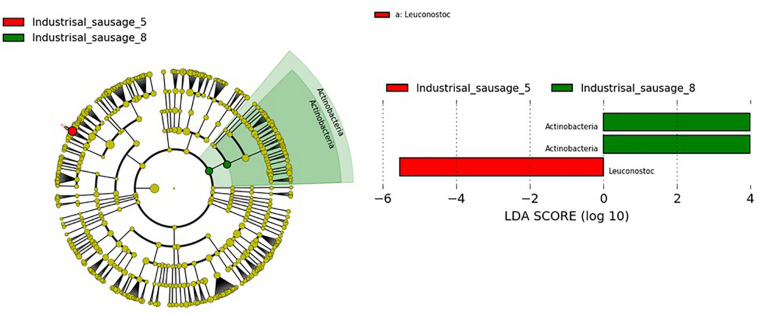
LEfSe analyses of taxon abundances of over-represented bacterial taxa among sausages.

Considering that beta diversity analyses revealed statistically significant groupings among traditional Pitsilia and industrial sausages, we performed statistical comparisons using the LEfSe algorithm to define the existence of differences in bacterial taxon abundances. The analysis showed enriched *Leuconostoc* in the industrial sausage 5 and Actinobacteria in the industrial sausage 8 (Figure 7).

## Discussion

Emerging evidence suggests that the microbial diversity of fermented products is differentiated based on the processing conditions and the area of production (Bokulich et al., 2016; Anagnostopoulos et al., 2019; Kamilari et al., 2019; Kamimura et al., 2019; van Reckem et al., 2020). In the present study, HTS was applied for a first in-depth quantitative characterization of Cyprus sausages’ bacterial diversity, mapping the variation in the bacterial consortia of traditional Pitsilia sausages compared to industrial ones. Nine different DNA isolation protocols were applied to evaluate the DNA extraction process’ contribution to the 16S rDNA sequencing results. The study revealed differences in both alpha and beta diversity among traditional and industrial sausages, suggesting that the bacterial communities may indicate a traditional product’s typicity.

Traditional Pitsilia sausages indicated increased alpha diversity (Shannon and Simpson indices) compared to the industrial sausages, except for sausage sample 6. Higher diversity in the traditional Pitsilia sausages could be explained by the absence of preservatives, in contrast to the industrial sausages. Preservatives such as nitrite, for instance, are added to increase safety and extend the shelf life of meat products due to their antimicrobial activity against pathogenic and/or spoilage bacteria (Majou and Christieans, 2018). Cardinali et al. (2018) indicated that increments in the concentration of nitrate during Fabriano-like fermented sausages production were negatively associated with bacterial communities’ richness and evenness. However, a recent study performed by Pini et al. (2020) identified no differences in the alpha diversity among dry-fermented sausages produced with sodium nitrite addition and with nitrite alternatives (grape seed extract, chestnut extract, and hydroxytyrosol extracted by olive pomace).

Additionally, the increased diversity in the traditional Pitsilia sausages could be attributed to the different processing conditions. Specifically, Pitsilia sausage producers apply maturation in a dry red wine while smoking using wood from indigenous trees or bushes as a preservation method. In contrast, the industry applies cold smoking and/or air heat, and sausages are usually cured but not fermented. In agreement with our findings, amplicon sequencing analysis revealed that Chinese dry-cured sausage had lower alpha diversity (Shannon index) and identified number of OTUs compared to Chinese smoke-cured sausage (Wang et al., 2018).

Our decision to apply nine different protocols for DNA isolation allowed us to identify the importance of the bacterial consortia’s contribution in defining the typicity of Pitsilia sausages. When performing a HTS analysis, the same protocol of sample and DNA processing, including sample homogenization, DNA extraction, primers used in the library preparation process, etc., has to be applied. Otherwise, differences in microbiota relative representation may appear. These differences may appear due to the different methodologies applied and not due to actual differences in the microbiota among samples. Independently of the applied protocol for DNA isolation, we observed distinct patterns of diversity in the bacterial community among Pitsilia traditional sausages and industrial sausages. The Bray–Curtis dissimilarity analysis showed that the similarity among sausages decreases significantly between Pitsilia sausages and industrial sausages. In contrast, a high similarity was observed among the sausage samples 8 and the Pitsilia sausage samples 2. Sausage samples 8 is claimed to be “traditional” although not complying with the PGI norms. This result could be potentially explained on the level of artisanal practices employed by each producer. Sausage samples 2 are produced in a recently renovated establishment, which potentially has limited artisanal established microbiota. These observations further support that the production site, in combination with the artisanal practices applied, contributes to the bacterial communities’ representation shaping.

Fermented sausage microbial consortia are differentiated based on their origin, raw material (the type of meat and fat content), minced meat processing, ingredients (salt concentration, spices and herbs, nitrate/nitrite, and other additives), size, the form of the casing, addition of starters, ripening conditions (relative humidity, temperature, the addition of molds, and smoke), as well as the environment (Hui and Sherkat, 2005; Leroy et al., 2013). OTU analysis of the 16S rRNA gene sequences revealed that Cyprus sausages’ core microbiota included LAB genera, mainly former *Lactobacillus* and *Leuconostoc* and in lower relative abundances *Pediococcus* and *Weissella*. During fermentation, LAB produce proteolytic enzymes responsible for the degradation of sarcoplasmic and myofibrillar proteins (Sanz and Toldrá, 2002; Fadda et al., 2010). They are considered key players in the meat fermentation and preservation process, via reducing the pH and producing bacteriocins able to prevent the proliferation of pathogens and contaminants. This increases the shelf life and the stability of the produced sausages (Fontana et al., 2005; Aquilanti et al., 2016). Several 16S rDNA amplicon sequencing studies in sausages confirmed their predominant relative abundance in sausages (Table 3). In contrast to our research, in which we identified a low number of reads corresponding to *Staphylococcus*, some researchers have observed their predominance (Wang et al., 2018; Chen et al., 2019; Pini et al., 2020). Comparison with the 16S rDNA metagenomic results from other smoke-cured sausages, such as the Spanish “Chorizo de Léon” (Quijada et al., 2018) and the Chinese “Lachang” (Wang et al., 2018), also indicated the predominant presence of *Lactobacillus* and *Pediococcus*, *Weissella*, as well as *Lactobacillus* and *Lactococcus* in lower abundances, respectively. These results support that some LAB strains contain the ability to tolerate and dominate the bacteria communities of sausages during smoking processing. The most representative species that the OTU analysis detected were *Lev. brevis* and *Com. paralimentarius.* Reads corresponding to the species *L. zeae* and *Leuc. mesenteroides* were also found. Due to their increased tolerance in salt and nitrite during fermentation, *Lev. brevis* and *Com. paralimentarius* are commonly found in fermented sausages, contributing to characteristic flavors and texture development in sausages (Borović et al., 2017; Xia et al., 2017; Reale et al., 2019). Statistical comparisons using the LEfSe algorithm showed significantly enriched *Leuconostoc* in the industrial sample 5. *Leuconostoc* species, such as *Leuc. mesenteroides*, are generally considered safe. Due to their beneficial contribution, which includes lactic acid, vitamins, bacteriocins, and exopolysaccharide (EPS) production, they are commonly used as starter cultures in the food industries (Stiles, 1994; Carr et al., 2002; Patel et al., 2012). However, under specific environmental conditions, including packaged refrigerated meat and dairy products (Diez et al., 2009; Comi et al., 2016), and high CO_2_ levels developed in long-term stored raw fruit and vegetables (Dror et al., 2019), *Leuconostoc* spp. act as spoilage bacteria. Their ability to survive pasteurization and grow under refrigerated conditions causing spoilage constitutes a serious concern for meat product industries (de Paula et al., 2015). Still, the exact conditions that influence the *Leuconostoc* growth remain undetermined. To overcome the detrimental consequences of *Leuc*. *mesenteroides*, Comi et al. (2016) suggested applying *Lactococcus lactis* and *Lat. sakei* as starter culture in commercial cooked bacon.

**TABLE 3:** Relative abundances of bacterial genera (and some species) that were detected in sausages via 16S rDNA sequencing.

**Type of sausage**	**Region**	**Relative abundance**			**Reference**
		**≥25%**	**10%–24%**	**1%–9%**	
Pitsilia	Cyprus	*Lactobacillus* and *Leuconostoc*		*Pseudomonas*, *Pediococcus*, *Weissella*, *Sediminibacterium*, *Acinetobacter*, and *Brochothrix*	Present study
Cacholeira blood-containing sausage	Portugal	*Lactobacillus* (*L*. *sakei*)		*Carnobacterium*, *Enterococcus*, *Kluyvera*, and *Serratia*	Belleggia et al. (2020)
Cinta Senese dry fermented	Italy	*Staphylococcus* (*S. xylosus*) and *Lactobacillus* (*L. sakei*)			Pini et al. (2020)
Game meat	Croatia	*Lactobacillus* (*L. sakei* and *L. curvatus*)	*Stenotrophomonas*	*Leuconostoc*, *Weissella*, *Lactococcus*, *Staphylococcus*, *Bacillus*, *Brochothrix*, *Pseudomonas*, and *Bradyrhizobium*	Mrkonjic Fuka et al. (2020) Bacterial diversity of naturally fermented game meat sausages: Sources of new starter cultures
Chorizo de Léon	Castilla y León (Spain)	*Lactobacillus* (*L. fuchuensis* and *L. curvatus*)	*Brochothrix*	*Staphylococcus* (*S. xylosus)* and *Leuconostoc, Bacillus*	Quijada et al. (2018)
Spanish-type “Chorizo”	Hidalgo, Mexico	*Lactobacillus*	*Streptococcus*	*Acinetobacter*, *Pseudomonas*, *Enterococcus*, *Brochothrix*, *Lactococcus*, and *Bacillus*	Juárez-Castelán et al. (2019)
Llama meat	Laguna de los Pozuelos, Argentina	*Lactobacillus* (*L. sakei* and *L. lactis*) and *Leuconostoc* (*L. inhae* and *Leuc. mesenteroides*)		*Staphylococcus* (*S. saprophyticus*)	Fontana et al. (2016)
	Northwest Argentina	*Lactobacillus* (*L. sakei*) and *Pseudomonas*	*Acinetobacter* and *Leuconostoc* (*L. inhae* and *Leuc. mesenteroides*)		
Cantonese	China	*Lactobacillus*	*Weissella*	*Pediococcus* and *Vibrio*	Wang et al. (2020)
Sichuan	China	*Lactobacillus* and *Weissella*		*Pediococcus*, *Brochothrix*, *Yersinia*, *Staphylococcus*, and *Enterobacter*	Wang et al. (2019)
Harbin	China	*Staphylococcus* and *Lactobacillus*		*Weissella*, *Acinetobacter*, Lachnospiraceae, *Alkalibacillus*, *Brochothrix*, *Macrococcus*, and *Coxiella*	Chen et al. (2019)
Dry-cured “Lachang”	China	*Staphylococcus*	*Acinetobacter*	*Weissella*, *Lactococcus*, *Aeromonas*, *Pseudomonas*, and *Photobacterium*	Wang et al. (2018)
Smoke-cured “Lachang”		*Weissella*	*Pediococcus*	*Lactobacillus*, *Lactococcus*, *Staphylococcus*, *Acinetobacter*, and *Psychrobacter*	

Apart from LAB, spoilage and contaminants, including *Pseudomonas*, represented by the species *P. umsongensis* and *P. alcaliphila*, *Acinetobacter*, *Brochothrix*, *Sedimentibacterium*, *Sphingomonas*, members of the family *Enterobacteriaceae*, etc. were also detected. The observed genera were widespread in the examined sausages but in different relative abundances. For instance, most samples from sausage sample 2 had increased relative representation of the genus *Sedimentibacterium* than most samples from the other sausages. However, a correlation analysis indicated that none could be identified as a biomarker for the specific sausage. These bacteria have been frequently identified in sausages. For instance, *Pseudomonas* and *Acinetobacter* were found to be among the predominant genera in “llama” sausages that originated from a plant in Northwest Argentina and dry-cured “Lachang” sausage that originated from China (Fontana et al., 2016; Wang et al., 2018). These genera were also identified using 16S rDNA metagenomic analysis in several other Cyprus products (Anagnostopoulos et al., 2020; Kamilari et al., 2020a, 2019; Papademas et al., 2020). *Pseudomonas* spp. possess the ability to create biofilms and adhere to the food processing plants’ equipment and surfaces, further supporting that these bacteria may be considered among the resident microbiota of the sausage processing environment. Furthermore, some species were detected in some sausages, such as *Staphylococcus saprophyticus*, whose presence has also been reported in llama meat by Fontana et al. (2016).

Estimating the impact of the nine different protocols of DNA extraction on the sausages’ bacterial community representation among sausages (beta diversity) revealed that most DNA samples that originated from the same sausage group were clustered together. Still, some protocols proved more efficient than others in the examined parameters. Specifically, DNA yield lower than 5 ng/μl is considered insufficient for the library preparation process’ first PCR reaction. Protocols such as the NS_B, NS_E, MB_B, and MB_E were more suitable for performing metagenomic analysis of the extracted bacterial DNA, compared to the protocols BP_B and BP_H. Still, none of the applied protocols sufficed to eliminate the presence of contaminants. Although the absorbance ratio 260/280 nm confirmed the purity of DNA for almost all isolates, the 260/230 nm revealed contaminants’ presence. Moreover, the NS_E method resulted in a broader diversity of bacterial communities using 16S rDNA sequencing. Surprisingly, this protocol was shown to be among the least effective in isolating total bacteria, *Lactobacillus*, and *Enterococcus* using qPCR. Additionally, the protocol MB_B that was the most efficient in the isolation of total bacteria, *Lactobacillus*, and *Enterococcus* using qPCR produced lower alpha diversity using 16S rDNA sequencing compared to the other protocols. Although the Pafos traditional sausage sample 4 showed lower alpha diversity compared to Pitsilia sausages, they indicated higher absolute copy number of total bacteria, *Lactobacillus*, *Enterococcus*, *Bacillus*, and ITS region via qPCR analysis. This observation highlights the importance of combining qPCR with alpha diversity analysis for more accurate microbial diversity characterization.

## Conclusion

Our metagenomic study is the first attempt performed to characterize Cyprus sausages’ bacterial diversity from the mountainous region of Pitsilia in small traditional cured meat-producing establishments (applying artisanal practices) and to reveal possible differences with sausages produced in large industrial establishments. The study revealed significantly higher bacterial alpha diversity between the traditional Pitsilia sausages and all but one industrial sausages by applying nine different DNA extraction protocols. Additionally, the total bacterial diversity (beta diversity) of each of the three Pitsilia sausages was clearly separated from the industrial sausages in all but one industrial sausage that also originated from Pitsilia. In the future, a higher number of different batches of the Pitsilia sausage-producing establishments and the industrially made sausages are to be analyzed by applying only one of the most efficient protocols of DNA isolation identified in the present study. Additionally, metagenomic may be combined with metabolomic analysis, to reveal the influence of the bacterial and fungal consortia in sausage flavor and color (Cruxen et al., 2019), or with isotopic or proteomic analysis, a method that is also applied to assess food authentication (Creydt and Fischer, 2018). These will further strengthen the present findings that the microbial patterns distinguish Pitsilia from the industrial sausages, providing a typicity impact that can potentially be used for authenticity control purposes. All these findings strengthen both the further improvement of quality characteristics as well as the marketing of Cyprus artisanal cured meats.

## Data Availability Statement

The datasets presented in this study can be found in online repository at https://www.ncbi.nlm.nih.gov/bioproject/PRJNA679907.

## Author Contributions

DT and EK were involved in the conceptualization of the study. EK, ME, and DA were involved in the methodology of the study. EK conducted the formal analysis, investigation, data curation, and writing and preparation of the original draft. DT was involved in the acquisition of resources, supervision, project administration, and funding acquisition. DT and DA were involved in writing the review and editing. All authors have read and agreed to the published version of the manuscript.

## Conflict of Interest

The authors declare that the research was conducted in the absence of any commercial or financial relationships that could be construed as a potential conflict of interest.

## Footnotes

1 https://cels.uri.edu/gsc/cndna.htmL

2 https://support.illumina.com/documents/documentation/chemistry_documentation/16s/16s-metagenomic-library-prep-guide-15044223-b.pdf

## Supplementary Material

Supplementary Figure 1 16S rRNA copy number and ITS standard curves and melting curves analyses from the real-time PCR assay. **(A)** V3V4 primers: The standard curve obtained from 10-fold serially diluted *Latilactobacillus sakei* pure genomic DNA. **(B)** *Lactobacillus* primers: The standard curve obtained from 10-fold serially diluted *Lat. sakei* pure genomic DNA. **(C)** *Bacillus* primers: The standard curve obtained from 10-fold serially diluted *Bacillus subtilis* pure genomic DNA. **(D)** *Enterococcus* primers: The standard curve obtained from 10-fold serially diluted *Enterococcus faecalis* pure genomic DNA. **(E)** ITS1 primers: The standard curve obtained from 10-fold serially diluted *Debaryomyces hansenii* pure genomic DNA. Measurements for each dilution were performed in triplicates.

Supplementary Figure 2 Exploration of alpha diversity based on the Simpson index in sausage samples. **(A)** Comparison of the different sausage samples. **(B)** Comparison of the different protocols of DNA extraction. Statistical analysis was performed using the Kruskal–Wallis test.

Supplementary Figure 3 The relative abundance of the 20 most abundant bacteria identified at the genus level based on 16S rDNA sequencing for the sausages: 1 **(A)**, 2 **(B)**, 3 **(C)**, 4 **(D)**, 5 **(E)**, 6 **(F)**, 7 **(G)**, and 8 **(H)**.

Supplementary Table 1 List of the primers, target genes, and qPCR analysis conditions.

Supplementary Table 2 Summary of the results of DNA extraction using blackPREP Food DNA I Kit, with the modification of bead treatment in the cell lysis step on the quantity and quality of DNA.

Supplementary Table 3 Summary of the results of DNA extraction using blackPREP Food DNA I Kit, with the modification of enzymatic treatment in the cell lysis step on the quantity and quality of DNA.

Supplementary Table 4 Summary of the results of DNA extraction using blackPREP Food DNA I Kit, with the modification of heat treatment in the cell lysis step on the quantity and quality of DNA.

Supplementary Table 5 Summary of the results of DNA extraction using DNeasy^®^ PowerFood^®^ Microbial Kit, with the modification of bead treatment in the cell lysis step on the quantity and quality of DNA.

Supplementary Table 6 Summary of the results of DNA extraction using DNeasy^®^ PowerFood^®^ Microbial Kit, with the modification of enzymatic treatment in the cell lysis step on the quantity and quality of DNA.

Supplementary Table 7 Summary of the results of DNA extraction using DNeasy^®^ PowerFood^®^ Microbial Kit, with the modification of heat treatment in the cell lysis step on the quantity and quality of DNA.

Supplementary Table 8 Summary of the results of DNA extraction using Nucleospin^®^ Food Kit, with the modification of bead treatment in the cell lysis step on the quantity and quality of DNA.

Supplementary Table 9 Summary of the results of DNA extraction using Nucleospin^®^ Food Kit, with the modification of enzymatic treatment in the cell lysis step on the quantity and quality of DNA.

Supplementary Table 10 Summary of the results of DNA extraction using Nucleospin< Food Kit, with the modification of thermal treatment in the cell lysis step on the quantity and quality of DNA.

Supplementary Table 11 Selected bacteria calculation of 16S rRNA copy number.

Supplementary Table 12 Information regarding identified number of reads, bacterial alpha diversity indexes, and observed OTU per analyzed sample.
