# Supplementary material for: Cyprus Sausages’ Bacterial Community Identification Through Metataxonomic Sequencing: Evaluation of the Impact of Different DNA Extraction Protocols on the Sausages’ Microbial Diversity Representation
Source: Front Microbiol. 2021 May 17;12:662957. doi: 10.3389/fmicb.2021.662957 (PMC8165277; doi:10.3389/fmicb.2021.662957)
Supplement: Supplementary Figure 1 — 16S rRNA copy number and ITS standard curves and melting curves analyses from the real-time PCR assay. (A) V3V4 primers: The standard curve obtained from 10-fold serially diluted Latilactobacillus sakei pure genomic DNA. (B) Lactobacillus primers: The standard curve obtained from 10-fold serially diluted Lat. sakei pure genomic DNA. (C) Bacillus primers: The standard curve obtained from 10-fold serially diluted Bacillus subtilis pure genomic DNA. (D) Enterococcus primers: The standard curve obtained from 10-fold serially diluted Enterococcus faecalis pure genomic DNA. (E) ITS1 primers: The standard curve obtained from 10-fold serially diluted Debaryomyces hansenii pure genomic DNA. Measurements for each dilution were performed in triplicates. [file Data_Sheet_1.PDF]

A

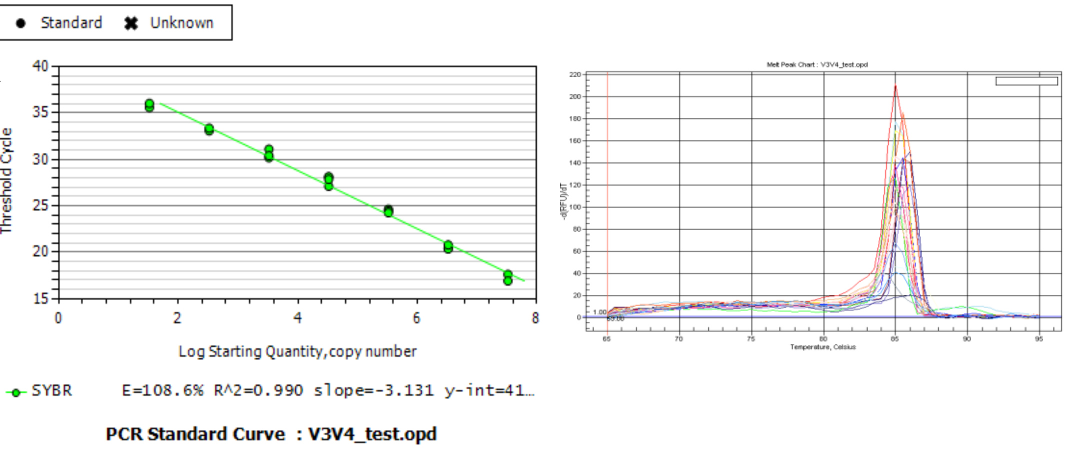

Standard
Unknown
Threshold Cycle
Log Starting Quantity, copy number
SYBR E=108.6% R^2=0.990 slope=-3.131 y-int=41...
PCR Standard Curve : V3V4_test.opd
Melt Peak Chart : V3V4_test.opd
-d(RFU)/dT
Temperature, Celsius

B.

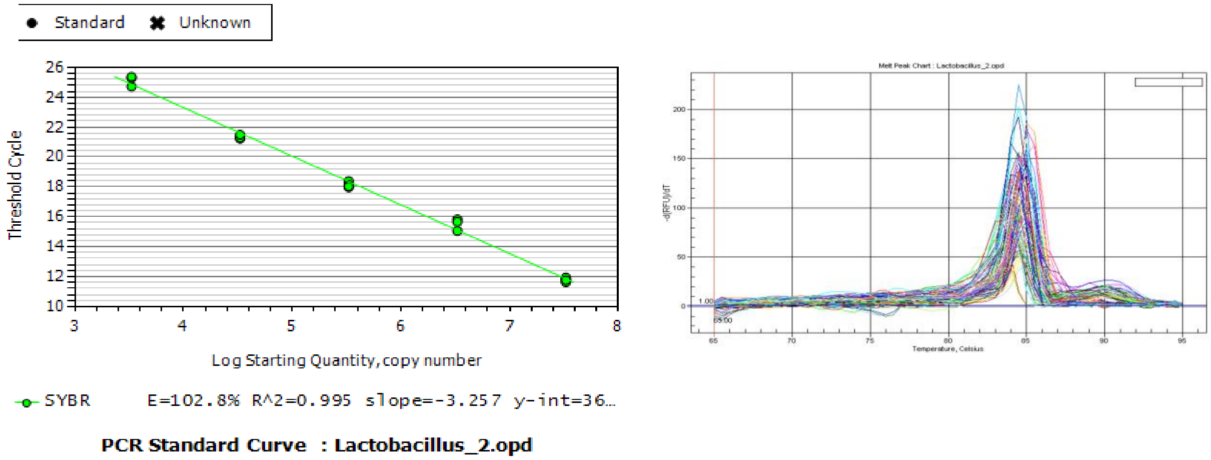

Standard
Unknown
Threshold Cycle
Log Starting Quantity, copy number
SYBR E=102.8% R^2=0.995 slope=-3.257 y-int=36...
PCR Standard Curve : Lactobacillus_2.opd
Melt Peak Chart : Lactobacillus_2.opd
-d(RFU)/dT
Temperature, Celsius

C.

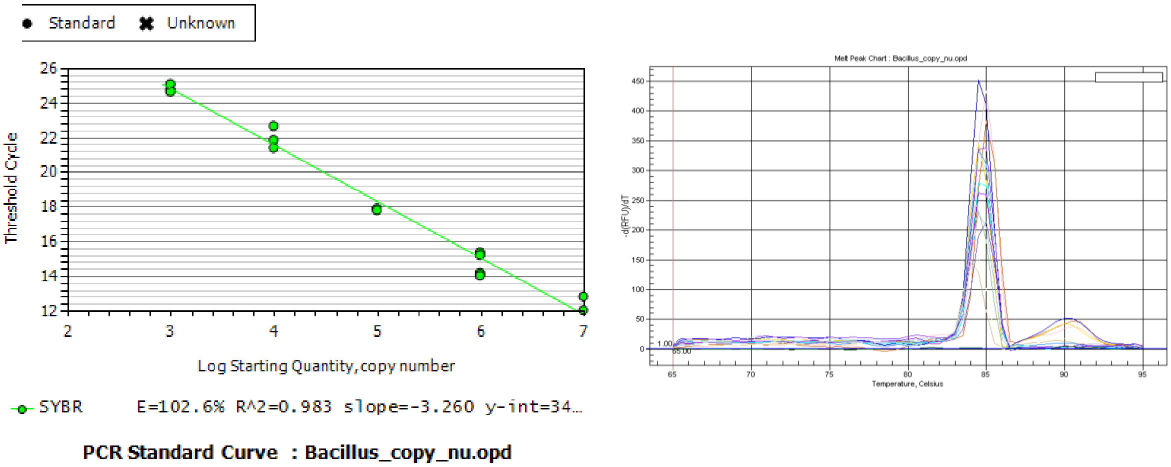

Standard
Unknown
Threshold Cycle
Log Starting Quantity, copy number
SYBR E=102.6% R^2=0.983 slope=-3.260 y-int=34...
PCR Standard Curve : Bacillus_copy_nu.opd
Melt Peak Chart : Bacillus_copy_nu.opd
-d(RFU)/dT
Temperature, Celsius

D.

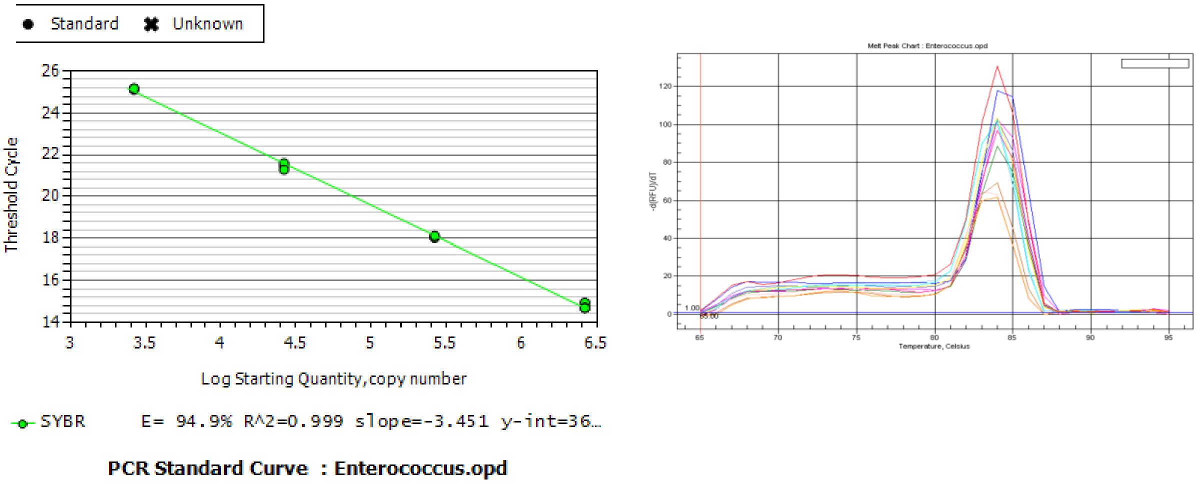

E.

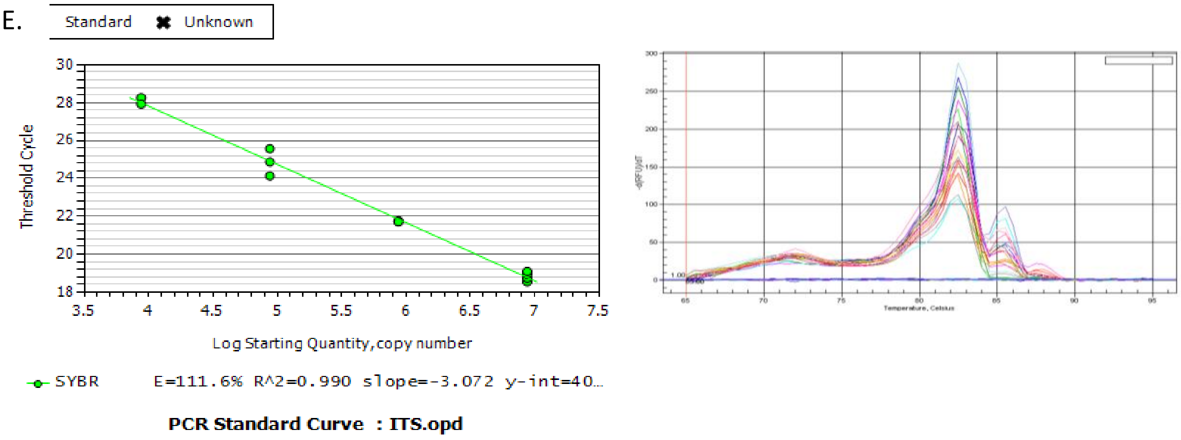

Figure S1. 16S rRNA copy number and ITS standard curves and melting curves analysis from the real time PCR assay. A. V3V4 primers: The standard curve obtained from 10-fold serially diluted *Lactobacillus sakei* pure genomic DNA. B. *Lactobacillus* primers: The standard curve obtained from 10-fold serially diluted *Lactobacillus sakei* pure genomic DNA. C: *Bacillus* primers: The standard curve obtained from 10-fold serially diluted *Bacillus subtilis* pure genomic DNA. D. *Enterococcus* primers: The standard curve obtained from 10-fold serially diluted *Enterococcus faecalis* pure genomic DNA. E: ITS1 primers: The standard curve obtained from 10-fold serially diluted *Debaryomyces.hansenii* pure genomic DNA. Measurements for each dilution were performed in triplicates.
