# Supplementary material for: Cyprus Sausages’ Bacterial Community Identification Through Metataxonomic Sequencing: Evaluation of the Impact of Different DNA Extraction Protocols on the Sausages’ Microbial Diversity Representation
Source: Front Microbiol. 2021 May 17;12:662957. doi: 10.3389/fmicb.2021.662957 (PMC8165277; doi:10.3389/fmicb.2021.662957)
Supplement: Supplementary Figure 2 — Exploration of alpha diversity based on the Simpson index in sausage samples. (A) Comparison of the different sausage samples. (B) Comparison of the different protocols of DNA extraction. Statistical analysis was performed using the Kruskal–Wallis test. [file Data_Sheet_2.PDF]

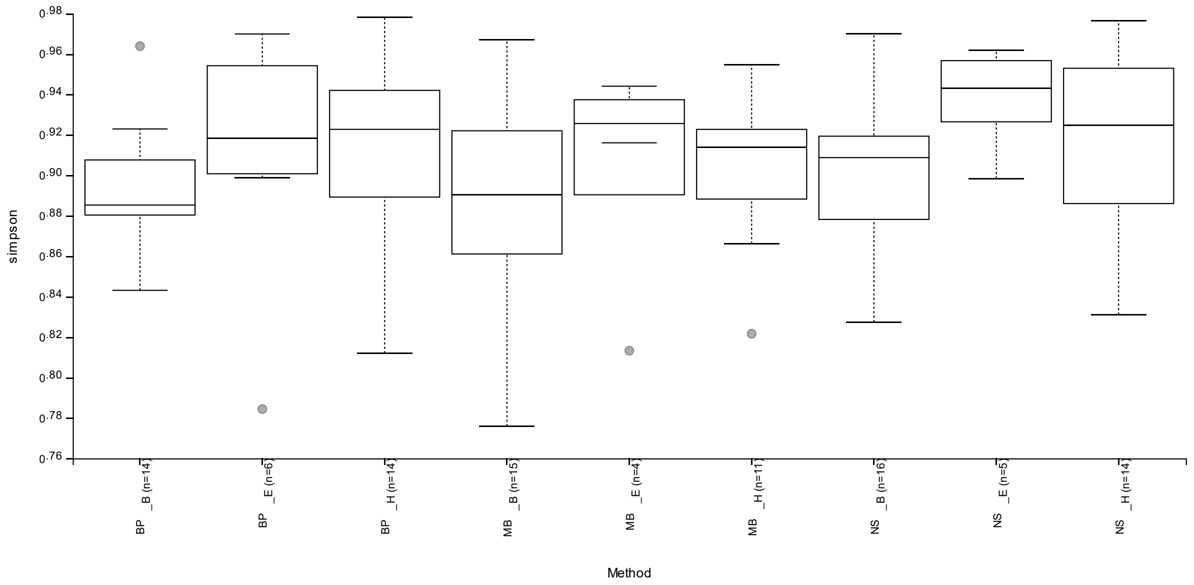

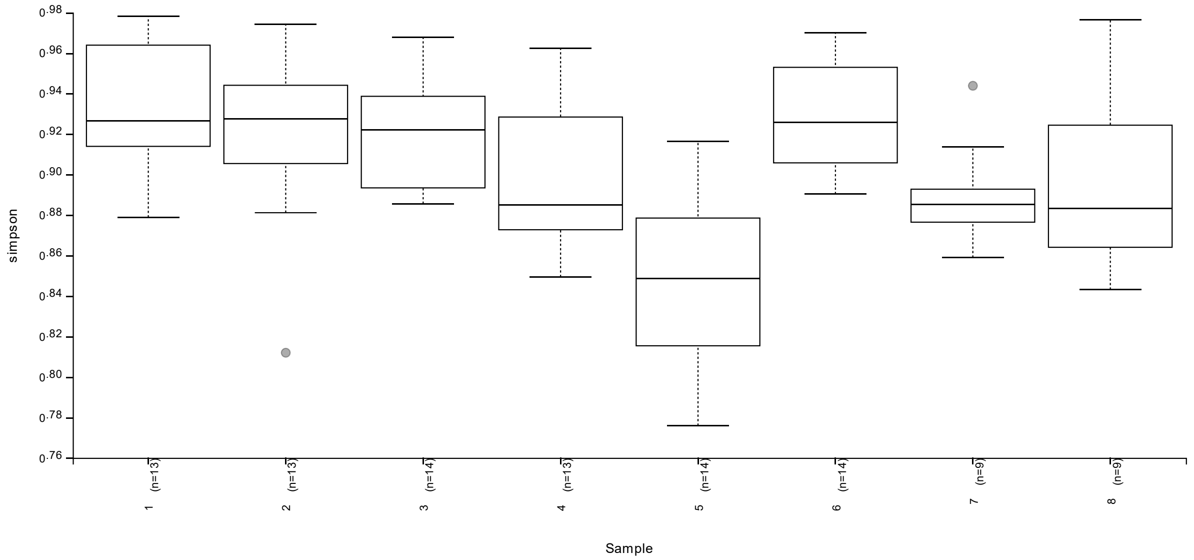

Figure S2. Exploration of alpha diversity based on the Simpson index in sausage samples. (A) Comparison of the different protocols of DNA extraction. (B) Comparison of the different sausages samples. Statistical analysis was performed using Kruskal-Wallis test.
