# Supplementary material for: Cyprus Sausages’ Bacterial Community Identification Through Metataxonomic Sequencing: Evaluation of the Impact of Different DNA Extraction Protocols on the Sausages’ Microbial Diversity Representation
Source: Front Microbiol. 2021 May 17;12:662957. doi: 10.3389/fmicb.2021.662957 (PMC8165277; doi:10.3389/fmicb.2021.662957)
Supplement: Supplementary Figure 3 — The relative abundance of the 20 most abundant bacteria identified at the genus level based on 16S rDNA sequencing for the sausages: 1 (A), 2 (B), 3 (C), 4 (D), 5 (E), 6 (F), 7 (G), and 8 (H). [file Data_Sheet_3.PDF]

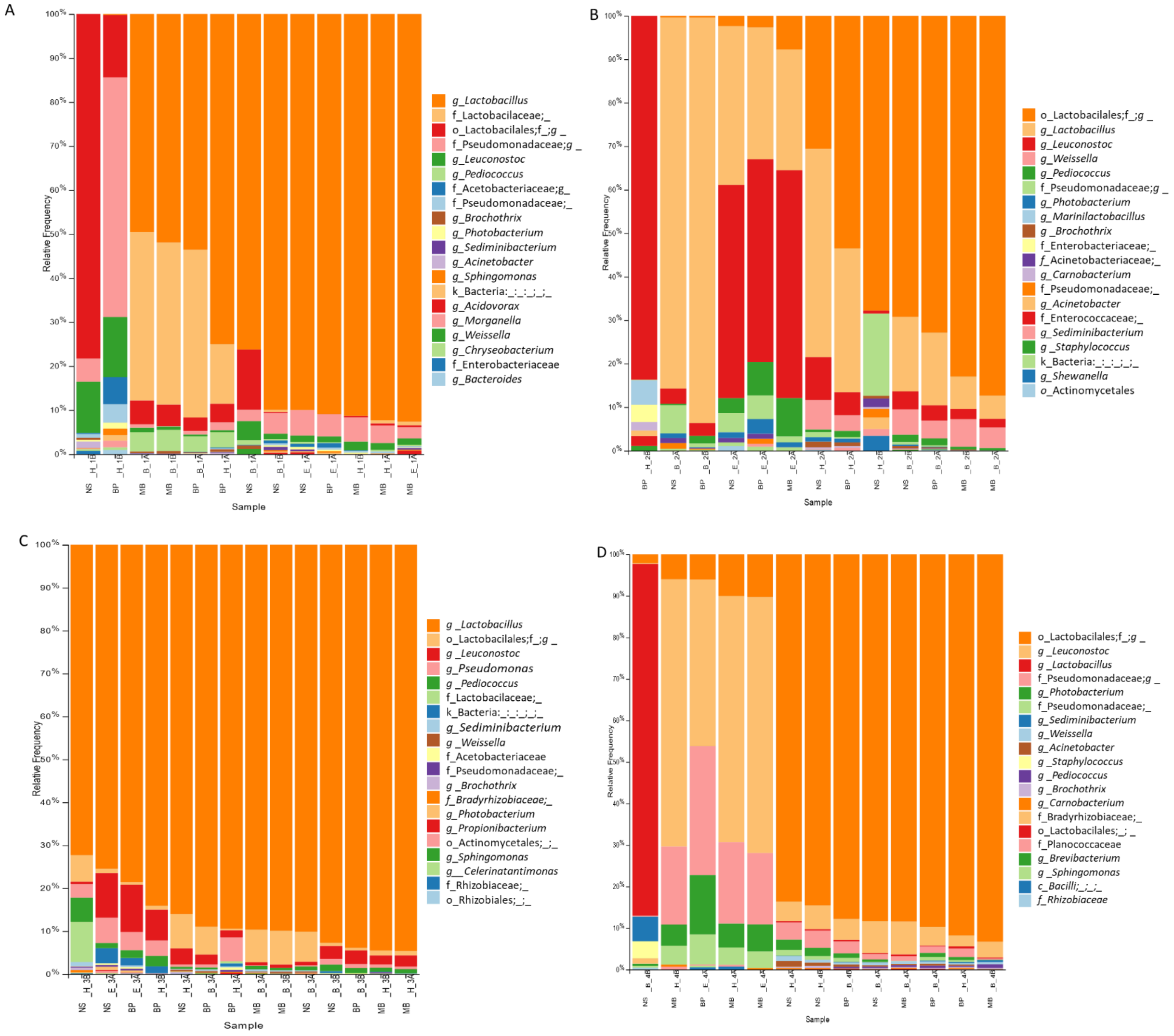
A
Relative Frequency
Sample
g_Lactobacillus
f_Lactobacilaceae;_
o_Lactobacilales;f_;g _
f_Pseudomonadaceae;g _
g_Leuconostoc
g_Pediococcus
f_Acetobacteriaceae;g_
f_Pseudomonadaceae;_
g_Brochothrix
g_Photobacterium
g_Sediminibacterium
g_Acinetobacter
g_Sphingomonas
k_Bacteria:_:_:_;_;_
g_Acidovorax
g_Morganella
g_Weissella
g_Chryseobacterium
f_Enterobacteriaceae
g_Bacteroides
B
Relative Frequency
Sample
o_Lactobacilales;f_;g _
g_Lactobacillus
g_Leuconostoc
g_Weissella
g_Pediococcus
f_Pseudomonadaceae;g _
g_Photobacterium
g_Marinilactobacillus
g _Brochothrix
f_Enterobacteriaceae;_
f_Acinetobacteriaceae;_
g_Carnobacterium
f_Pseudomonadaceae;_
g_Acinetobacter
f_Enterococcaceae;_
g_Sediminibacterium
g _Staphylococcus
k_Bacteria:_:_:_;_;_
g_Shewanella
o_Actinomycetales
C
Relative Frequency
Sample
g _Lactobacillus
o_Lactobacilales;f_;g _
g _Leuconostoc
g_Pseudomonas
g _Pediococcus
f_Lactobacilaceae;_
k_Bacteria:_:_:_;_;_
g_Sediminibacterium
g _Weissella
f_Acetobacteriaceae
f_Pseudomonadaceae;_
g _Brochothrix
f_Bradyrhizobiaceae;_
g_Photobacterium
g_Propionibacterium
o_Actinomycetales;_;_
g_Sphingomonas
g__Celerinatantimonas
f_Rhizobiaceae;_
o_Rhizobiales;_;_
D
Relative Frequency
Sample
o_Lactobacilales;f_;g _
g _Leuconostoc
g _Lactobacillus
f_Pseudomonadaceae;g _
g_Photobacterium
f_Pseudomonadaceae;_
g_Sediminibacterium
g_Weissella
g_Acinetobacter
g _Staphylococcus
g _Pediococcus
g _Brochothrix
g_Carnobacterium
f_Bradyrhizobiaceae;_
o_Lactobacilales;_; _
f_Planococcaceae
g_Brevibacterium
g _Sphingomonas
c_Bacilli;_;_;_
f_Rhizobiaceae

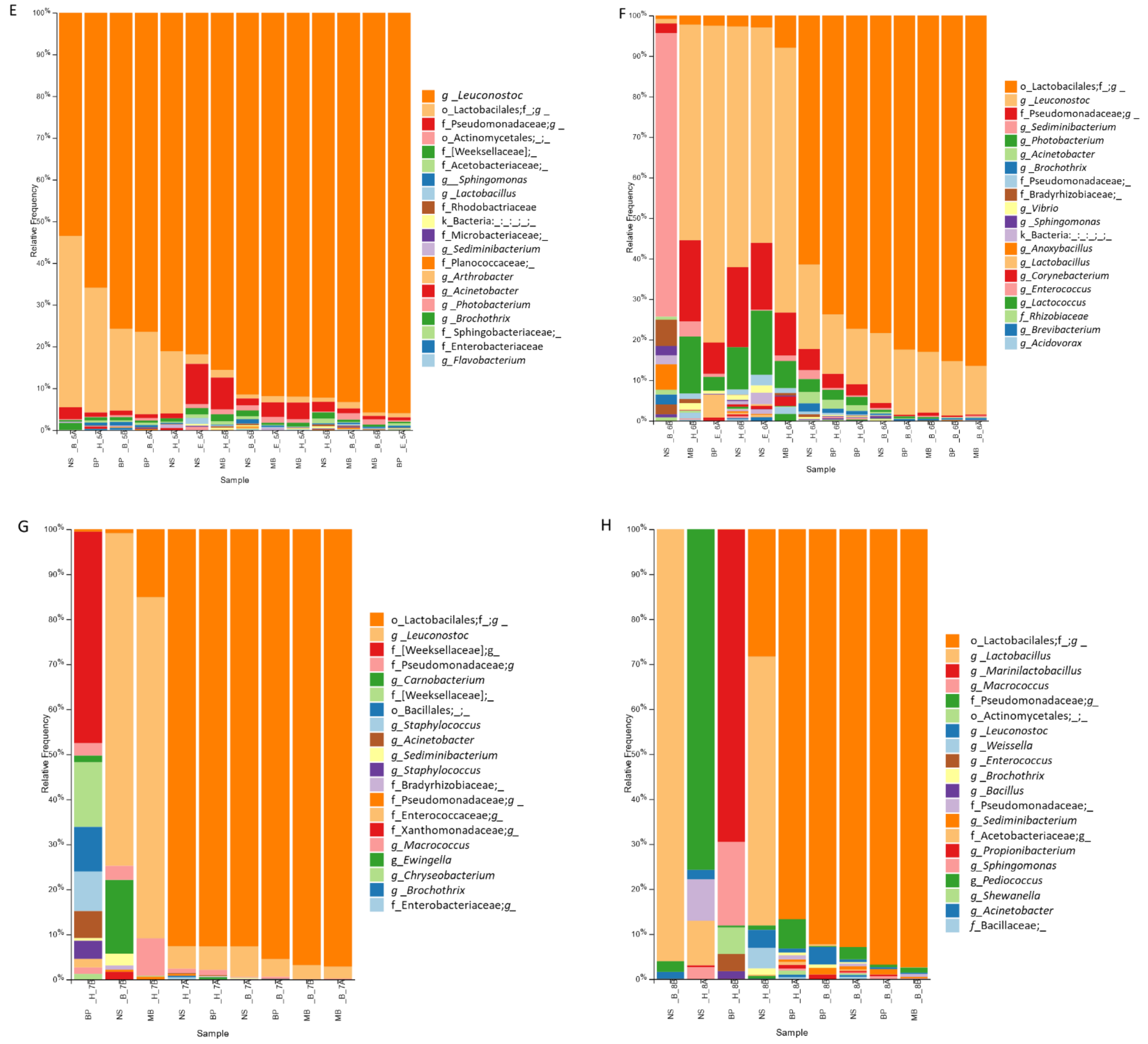

Figure S3. The relative abundance of the twenty most abundant bacteria identified at the genus level based on 16S rDNA sequencing for the sausages: 1 (A), 2 (B), 3 (C), 4 (D), 5 (E), 6 (F), 7 (G) and 8 (H).
