# Supplementary material for: Cyprus Sausages’ Bacterial Community Identification Through Metataxonomic Sequencing: Evaluation of the Impact of Different DNA Extraction Protocols on the Sausages’ Microbial Diversity Representation
Source: Front Microbiol. 2021 May 17;12:662957. doi: 10.3389/fmicb.2021.662957 (PMC8165277; doi:10.3389/fmicb.2021.662957)
Supplement: Supplementary Table 1 — List of the primers, target genes, and qPCR analysis conditions. [file Table_1.docx]

**Table S1**. List of the primers, target genes and qPCR analysis conditions.

| Primers name | Sequence | Target region | Standard curve reference species | qPCR conditions | Reference |
| --- | --- | --- | --- | --- | --- |
| V3V4 | Forward: 5’- CCTACGGGRGGCAGCAG-3’ | V3V4 region of the 16S rRNA gene | *B. subtilis* | 50°C for 2 min; 95°C for 3 min; followed by 40 cycles of 30 seconds at 95°C and 30 s minute at 60°C | (Klindworth *et al.*, 2013) |
|  | Reverse: 5’-GACTACHVGGGTATCTAATCC-3’ |  |  |  |  |
| *Enterococcus* | Forward primer: 5'-GGACGAAAGTCTGACCGA-3’ | 16S rRNA gene | *E.faecalis* | 50°C for 2 min; 95°C for 3 min; followed by 40 cycles of 30 seconds at 95°C and 30 s minute at 60°C | (Ryu *et al.*, 2013) |
|  | Reverse primer: 5'- TTAAGAAACCGCCTGCGC-3’ |  |  |  |  |
| *Bacillus* | Forward: 5’- ACGCCGTAAACGATGAGT-3’ | 16S rRNA gene | *B. subtilis* | 50°C for 2 min; 95°C for 3 min; followed by 40 cycles of 30 seconds at 95°C and 30 s minute at 60°C | (Han *et al.*, 2012) |
|  | Reverse: 5’-GTGTGTAGCCCAGGTCATAA-3’ |  |  |  |  |
| *Lactobacillus* | Forward: 5’- AGCAGTAGGGAATCTTCCA-3’ | 16S rRNA gene | *L. sakei* | 95°C for 30 sec; followed by 40 cycles of 10 seconds at 95°C and 30 s minute at 62°C | (Louise Kristine Vigsnæs, et al. 2011, Heilig H. G. H. J., et al. 2002) |
|  | Reverse: 5’- CACCGCTACACATGGAG-3’ |  |  |  |  |
| ITS1 | Forward: 5’- CTACCTGCGGARGGATCA-3’ | ITS1 | *D. hansenii* | 50°C for 2 min; 95°C for 3 min; followed by 40 cycles of 30 seconds at 95°C and 30 s minute at 57°C | (Bokulich *et al.*, 2016) |
|  | Reverse: 5′- GAGATCCRTTGYTRAAAGTT-3′ |  |  |  |  |

**Table S2**. Summary of the results of DNA extraction using blackPREP Food DNA I Kit, with the modification of bead treatment in the cell lysis step on the quantity and quality of DNA

|  | Results of DNA extraction from sausages | | | | | | | | | | |
| --- | --- | --- | --- | --- | --- | --- | --- | --- | --- | --- | --- |
| Sample ID | | NanoDrop | | | Qubit  ng/μl |  | | Copy Number | | |  |
|  |  | ng/μl | 260/280 | 260/230 |  | V3V4 | *Lactobacillus* | | *Bacillus* | *Enterococcus* | ITS |
| 01ABP_B | | 14,5 | 1,87 | 0,04 | 2.78 | 1.014E+07 | 8.944E+06 | | 1.72E+05 | 7,583E+03 | 5.529E+04 |
| 01BBP_B | | 8,6 | 1,7 | 0,22 | 1.73 | 2.154E+07 | 1.099E+07 | | 8.65E+05 | 7,897E+03 | 5.969E+05 |
| 02ABP_B | | 10,9 | 1,59 | 0,08 | 1.36 | 1.071E+07 | 5.602E+06 | | 9.58E+05 | 1,199E+04 | 1.401E+06 |
| 02BBP_B | | 11,5 | 1,54 | 0,45 | 0.98 | 7.440E+06 | 4.427E+06 | | 8.96E+05 | 4,114E+03 | 1.830E+06 |
| 03ABP_B | | 43,9 | 1,69 | 0,67 | 6.16 | 6.669E+06 | 1.006E+07 | | 1.99E+05 | 1,820E+03 | 9.206E+05 |
| 03BBP_B | | 10,3 | 2,2 | 0,18 | 4.12 | 2.723E+06 | 7.468E+06 | | 3.33E+06 | 2,340E+03 | 1.273E+06 |
| 04ABP_B | | 41,1 | 1,7 | 0,54 | 3.7 | 4.567E+06 | 9.700E+06 | | 2.53E+06 | 2,014E+04 | 1.287E+06 |
| 04BBP_B | | 52,1 | 1,54 | 0,39 | 8.46 | 7.461E+06 | 1.061E+07 | | 2.36E+06 | 1,414E+04 | 9.929E+05 |
| 05ABP_B | | 19,5 | 1,85 | 0,45 | 2.08 | 3.213E+06 | 2.136E+06 | | 6.802E+04 | 1,102E+03 | 4.279E+05 |
| 05BBP_B | | 4,6 | 2,8 | 0,15 | 1.54 | 1.612E+06 | 1.002E+06 | | 5.137E+04 | 2,761E+02 | 2.635E+05 |
| 06ABP_B | | 8,2 | 1,64 | 0,22 | 2.16 | 2.546E+07 | 2.011E+07 | | 4.213E+06 | 2,375E+04 | 1.02E+05 |
| 06BBP_B | | 8,8 | 1,54 | 0,23 | 1.65 | 1.479E+07 | 7.941E+06 | | 1.384E+06 | 1,198E+04 | 5.15E+04 |
| 07ABP_B | | 8 | 1,63 | 0,25 | 1.36 | 1.871E+06 | 6.307E+06 | | 8.930E+05 | 1,654E+03 | 2.683E+05 |
| 07BBP_B | | 5,5 | 1,89 | 0,1 | 0.5 | 1.458E+07 | 8.573E+06 | | 3.854E+04 | 2,788E+02 | 8.202E+04 |
| 08ABP_B | | 10,4 | 1,46 | 0,15 | 0.3 | 2.027E+04 | 2.352E+04 | | 2.114E+03 | 7,325E+01 | 6.368E+05 |
| 08BBP_B | | 5,1 | 1,14 | 0,05 | 0.5 | 4.806E+04 | 2.258E+04 | | 1.968E+03 | 1,613E+02 | 3.017E+05 |

**Table S3**. Summary of the results of DNA extraction using blackPREP Food DNA I Kit, with the modification of enzymatic treatment in the cell lysis step on the quantity and quality of DNA

|  | Results of DNA extraction from sausages | | | | | | | | | |
| --- | --- | --- | --- | --- | --- | --- | --- | --- | --- | --- |
| Sample ID | | NanoDrop | | | Qubit |  | Copy Number | | |  |
|  |  | ng/μl | 260/280 | 260/230 |  | V3V4 | *Lactobacillus* | *Bacillus* | *Enterococcus* | ITS |
| 01ABP_E | | 14 | 1,6 | 0,35 | 6.34 | 5.628E+06 | 5.181E+06 | 1.160E+06 | 1,198E+04 | 5.560E+05 |
| 01BBP_E | | 17 | 1,65 | 0,24 | 6.21 | 6.525E+06 | 9.231E+06 | 1.015E+06 | 9,958E+03 | 6.626E+05 |
| 02ABP_E | | 21,1 | 1,65 | 0,57 | 6.14 | 1.408E+06 | 7.936E+05 | 1.488E+05 | 7,562E+03 | 4.563E+05 |
| 02BBP_E | | 35 | 1,38 | 0,51 | 6.03 | 1.427E+06 | 6.168E+05 | 1.368E+05 | 4,610E+03 | 3.724E+05 |
| 03ABP_E | | 13,4 | 1,58 | 0,39 | 5.52 | 3.131E+06 | 2.516E+06 | 1.296E+06 | 3,528E+03 | 4.136E+05 |
| 03BBP_E | | 18,5 | 1.53 | 0,48 | 5.55 | 3.016E+06 | 1.506E+06 | 7.419E+05 | 2,976E+03 | 2.033E+05 |
| 04ABP_E | | 21 | 1,73 | 0,22 | 3.8 | 5.167E+06 | 4.604E+06 | 1.280E+06 | 9,813E+03 | 9.006E+06 |
| 04BBP_E | | 17,4 | 1,54 | 0,39 | 3.5 | 7.511E+06 | 2.531E+06 | 1.488E+06 | 1,460E+04 | 9.386E+06 |
| 05ABP_E | | 5,7 | 1,62 | 0,45 | 1.81 | 2.973E+06 | 6.171E+06 | 1.562E+05 | 2,103E+03 | 5.958E+05 |
| 05BBP_E | | 4,5 | 1,97 | 0,36 | 1.72 | 4.385E+06 | 2.221E+06 | 1.366E+05 | 7,526E+02 | 8.077E+05 |
| 06ABP_E | | 9,6 | 1,98 | 0,41 | 2.56 | 1.163E+06 | 7.402E+05 | 2.498E+05 | 2,031E+04 | 8.586E+05 |
| 06BBP_E | | 10,8 | 1,64 | 0,42 | 2.71 | 1.292E+06 | 5.973E+06 | 1.935E+05 | 1,097E+04 | 2.332E+06 |
| 07ABP_E | | 11,2 | 1,63 | 0,48 | 0.32 | 1.654E+05 | 1.267E+05 | 2.200E+04 | 2,092E+02 | 1.171E+06 |
| 07BBP_E | | 10,1 | 1,6 | 0,47 | 0.35 | 2.161E+05 | 1.316E+05 | 6.943E+03 | 1,190E+02 | 1.362E+06 |
| 08ABP_E | | 8,6 | 1,81 | 0,15 | 0.73 | 6.609E+04 | 1.944E+04 | 3.315E+03 | 1,342E+03 | 1.424E+06 |
| 08BBP_E | | 7,3 | 1,87 | 0,06 | 0.69 | 6.904E+04 | 1.173E+04 | 1.698E+03 | 1,078E+03 | 8.286E+05 |

**Table S4**. Summary of the results of DNA extraction using blackPREP Food DNA I Kit, with the modification of heat treatment in the cell lysis step on the quantity and quality of DNA

|  | Results of DNA extraction from sausages | | | | | | | | |
| --- | --- | --- | --- | --- | --- | --- | --- | --- | --- |
| Sample ID | NanoDrop | | | Qubit |  | Copy Number | | | |
|  | ng/μl | 260/280 | 260/230 | ng/μl | V3V4 | *Lactobacillus* | *Bacillus* | *Enterococcus* | ITS |
| 01ABP_H | 6,2 | 2,02 | 0,41 | 1.33 | 1.735E+07 | 1.586E+07 | 7.160E+05 | 1,296E+04 | 7.584E+05 |
| 01BBP_H | 10,3 | 1,79 | 0,16 | 0.95 | 4.382E+06 | 4.979E+06 | 1.838E+05 | 2,945E+03 | 2.347E+05 |
| 02ABP_H | 65,7 | 1,45 | 0,27 | 3.92 | 1.685E+06 | 1.868E+06 | 1.575E+05 | 1,152E+04 | 1.13E+03 |
| 02BBP_H | 14,7 | 1,77 | 0,21 | 1.17 | 6.341E+06 | 2.722E+06 | 4.017E+05 | 8,787E+03 | 8.60E+03 |
| 03ABP_H | 22,5 | 1,99 | 0,26 | 1.64 | 7.448E+06 | 5.155E+06 | 4.465E+05 | 2,556E+03 | 1.224E+06 |
| 03BBP_H | 17,9 | 1,97 | 0,42 | 5.5 | 4.365E+06 | 4.662E+06 | 1.121E+05 | 5,734E+02 | 6.833E+05 |
| 04ABP_H | 16,8 | 1,74 | 0,2 | 3.2 | 7.927E+06 | 7.641E+06 | 1.845E+05 | 1,077E+04 | 8.207E+05 |
| 04BBP_H | 18,2 | 1,87 | 0,23 | 1.86 | 9.211E+06 | 2.518E+06 | 4.398E+05 | 1,380E+04 | 2.515E+06 |
| 05ABP_H | 4,4 | 1,42 | 0,03 | 0.84 | 1.532E+06 | 1.638E+06 | 5.193E+04 | 1,840E+03 | 1.020E+06 |
| 05BBP_H | 5,9 | 1,41 | 0,05 | 0.3 | 1.249E+06 | 1.102E+06 | 4.971E+05 | 1,135E+03 | 6.544E+05 |
| 06ABP_H | 6 | 1,72 | 0,24 | 0.3 | 9.317E+05 | 6.945E+05 | 5.746E+04 | 1,298E+04 | 7.182E+05 |
| 06BBP_H | 4,2 | 1,94 | 0,10 | 0.86 | 1.058E+06 | 4.992E+05 | 6.520E+04 | 1,051E+04 | 6.678E+05 |
| 07ABP_H | 3,1 | 1,92 | 0,08 | 0.89 | 5.527E+06 | 3.226E+06 | 2.086E+05 | 2,001E+03 | 2.219E+05 |
| 07BBP_H | 7,3 | 1,81 | 0,13 | 0.9 | 2.181E+07 | 1.341E+07 | 1.655E+05 | 1,871E+03 | 5.175E+04 |
| 08ABP_H | 3 | 1,45 | 0,09 | 0.3 | 2.408E+05 | 9.454E+04 | 9.982E+04 | 7,691E+02 | 2.66E+03 |
| 08BBP_H | 2,7 | 1,06 | 0,06 | 0.47 | 1.003E+05 | 1.685E+05 | 2.006E+04 | 1,169E+03 | 4.06E+03 |

**Table S5**. Summary of the results of DNA extraction using DNeasy® PowerFood® Microbial Kit, with the modification of bead treatment in the cell lysis step on the quantity and quality of DNA

|  | Results of DNA extraction from sausages | | | | | | | | | | |
| --- | --- | --- | --- | --- | --- | --- | --- | --- | --- | --- | --- |
| Sample ID | | NanoDrop | | | Qubit  ng/μl |  | | Copy Number | | |  |
|  |  | ng/μl | 260/280 | 260/230 |  | V3V4 | *Lactobacillus* | | *Bacillus* | *Enterococcus* | ITS |
| 01AMB_B | | 7,2 | 1,59 | 0,7 | 6.02 | 4.915E+07 | 2.231E+07 | | 3.320E+06 | 1,754E+04 | 6.361E+04 |
| 01BMB_B | | 8,7 | 1,61 | 0,41 | 4.62 | 4.609E+07 | 2.044E+07 | | 2.733E+06 | 1,049E+04 | 4.137E+04 |
| 02AMB_B | | 10,2 | 1,8 | 1,31 | 8 | 4.467E+07 | 2.557E+07 | | 3.091E+06 | 1,931E+04 | 1.779E+06 |
| 02BMB_B | | 10,3 | 1,69 | 1,17 | 4.48 | 5.563E+07 | 4.739E+07 | | 4.631E+06 | 3,144E+04 | 3.396E+06 |
| 03AMB_B | | 30 | 1,82 | 0,31 | 12.6 | 4.066E+07 | 6.440E+07 | | 2.574E+06 | 3,479E+03 | 1.242E+06 |
| 03BMB_B | | 12,8 | 1,82 | 0,8 | 6.18 | 1.264E+07 | 1.846E+07 | | 8.833E+06 | 2,318E+04 | 4.286E+05 |
| 04AMB_B | | 11,3 | 1,94 | 1,17 | 11.1 | 8.820E+07 | 6.079E+08 | | 9.830E+07 | 2,160E+05 | 1.246E+07 |
| 04BMB_B | | 12 | 1,86 | 0,73 | 5 | 6.632E+07 | 4.720E+07 | | 9.459E+06 | 8,275E+04 | 3.179E+06 |
| 05AMB_B | | 6,1 | 2,45 | 0,05 | 4.7 | 1.564E+07 | 7.759E+06 | | 1.497E+05 | 3,882E+03 | 2.755E+05 |
| 05BMB_B | | 5,6 | 2,23 | 0,45 | 1.75 | 1.030E+07 | 7.196E+06 | | 3.753E+05 | 3,358E+03 | 6.463E+04 |
| 06AMB_B | | 12,1 | 1,91 | 0,83 | 14.2 | 1.387E+07 | 1.193E+07 | | 3.141E+06 | 7,983E+04 | 2.746E+07 |
| 06BMB_B | | 15,3 | 2,2 | 0,42 | 10.6 | 4.285E+07 | 1.990E+07 | | 2.342E+06 | 1,162E+05 | 2.006E+07 |
| 07AMB_B | | 3,8 | 1,65 | 0,18 | 8.32 | 4.764E+07 | 2.029E+07 | | 4.552E+06 | 2,647E+03 | 3.135E+04 |
| 07BMB_B | | 5,3 | 2,2 | 0,11 | 2.56 | 3.429E+07 | 1.573E+07 | | 3.914E+06 | 5,572E+03 | 4.968E+04 |
| 08AMB_B | | 5,8 | 2,02 | 0,13 | 4.94 | 4.520E+06 | 1.177E+06 | | 2.534E+06 | 8,726E+03 | 6.262E+04 |
| 08BMB_B | | 6 | 1,73 | 0,1 | 2.52 | 4.450E+06 | 1.908E+06 | | 7.447E+05 | 4,910E+03 | 1.472E+05 |

**Table S6**. Summary of the results of DNA extraction using DNeasy® PowerFood® Microbial Kit, with the modification of enzymatic treatment in the cell lysis step on the quantity and quality of DNA

|  | Results of DNA extraction from sausages | | | | | | | | | | |
| --- | --- | --- | --- | --- | --- | --- | --- | --- | --- | --- | --- |
| Sample ID | | NanoDrop | | | Qubit  ng/μl |  | | Copy Number | | |  |
|  |  | ng/μl | 260/280 | 260/230 |  | V3V3 | *Lactobacillus* | | *Bacillus* | *Enterococcus* | ITS |
| 01AMB_E | | 15,8 | 1,71 | 0,95 | 18.6 | 7.933E+06 | 5.151E+06 | | 1.225E+06 | 4,747E+03 | 2.311E+05 |
| 01BMB_E | | 14,5 | 1,8 | 1,1 | 17.8 | 1.191E+07 | 9.226E+06 | | 1.028E+06 | 1,513E+03 | 1.257E+05 |
| 02AMB_E | | 7,9 | 1,87 | 0,5 | 4.68 | 3.374E+07 | 2.162E+07 | | 9.656E+06 | 1,350E+05 | 6.985E+06 |
| 02BMB_E | | 6,2 | 1,85 | 0,55 | 4.71 | 5.107E+07 | 1.460E+07 | | 8.332E+06 | 5,566E+04 | 2.270E+06 |
| 03AMB_E | | 27,7 | 1,94 | 0,92 | 26.4 | 6.920E+06 | 5.372E+06 | | 9.541E+05 | 3,756E+02 | 4.539E+04 |
| 03BMB_E | | 28 | 2,1 | 0,91 | 25.3 | 7.906E+06 | 6.947E+06 | | 1.088E+06 | 1,695E+04 | 1.568E+06 |
| 04AMB_E | | 5,6 | 1,8 | 0,15 | 2.72 | 5.143E+07 | 4.220E+08 | | 4.727E+06 | 2,558E+04 | 4.22E+05 |
| 04BMB_E | | 6,2 | 1,9 | 0,2 | 1.8 | 6.977E+07 | 4.652E+07 | | 8.838E+06 | 1,656E+05 | 3.17E+05 |
| 05AMB_E | | 12,2 | 1,82 | 0,47 | 14.4 | 2.596E+06 | 9.133E+05 | | 6.940E+04 | 5,110E+03 | 9.765E+04 |
| 05BMB_E | | 11,6 | 1,8 | 0,51 | 9.7 | 2.091E+06 | 1.192E+06 | | 7.183E+04 | 2,779E+03 | 1.431E+05 |
| 06AMB_E | | 10,2 | 2,99 | 0,2 | 13.6 | 1.069E+07 | 6.659E+06 | | 7.265E+05 | 1,318E+04 | 8.831E+05 |
| 06BMB_E | | 8,4 | 2,1 | 0,23 | 12.1 | 3.581E+06 | 7.005E+06 | | 7.949E+05 | 1,446E+04 | 1.142E+06 |
| 07AMB_E | | 7,2 | 1,75 | 0,22 | 5.5 | 6.350E+06 | 7.016E+06 | | 9.718E+05 | 3,369E+03 | 6.321E+04 |
| 07BMB_E | | 6,5 | 1,65 | 0,25 | 4.7 | 6.432E+06 | 6.419E+06 | | 1.322E+06 | 2,489E+04 | 6.718E+04 |
| 08AMB_E | | 10 | 1,99 | 0,25 | 12.3 | 3.101E+05 | 1.147E+05 | | 2.276E+04 | 6,530E+03 | 1.811E+04 |
| 08BMB_E | | 9,1 | 1,92 | 0,24 | 10.6 | 1.966E+05 | 1.073E+05 | | 1.500E+04 | 1,923E+04 | 1.867E+04 |

**Table S7**. Summary of the results of DNA extraction using DNeasy® PowerFood® Microbial Kit, with the modification of heat treatment in the cell lysis step on the quantity and quality of DNA

|  | Results of DNA extraction from sausages | | | | | | | | | | |
| --- | --- | --- | --- | --- | --- | --- | --- | --- | --- | --- | --- |
| Sample ID | | NanoDrop | | | Qubit  ng/μl |  | | Copy Number | | |  |
|  |  | ng/μl | 260/280 | 260/230 |  | V3V4 | *Lactobacillus* | | *Bacillus* | *Enterococcus* | ITS |
| 01AMB_H | | 10,8 | 1,97 | 0,33 | 2.74 | 3.522E+06 | 2.050E+07 | | 2.863E+06 | 1,187E+04 | 8.139E+04 |
| 01BMB_H | | 25,5 | 2 | 0,83 | 3.92 | 1.539E+06 | 2.014E+07 | | 3.513E+06 | 4,392E+03 | 7.925E+04 |
| 02AMB_H | | 16,9 | 2,15 | 1,07 | 0.5 | 2.921E+06 | 6.192E+06 | | 4.701E+06 | 1,525E+04 | 9.511E+05 |
| 02BMB_H | | 13,7 | 2,11 | 0,2 | 3.62 | 2.851E+06 | 1.861E+07 | | 2.884E+06 | 6,977E+03 | 2.089E+06 |
| 03AMB_H | | 17,7 | 1,97 | 1,41 | 0.76 | 2.751E+06 | 3.426E+07 | | 4.833E+06 | 4,327E+03 | 9.343E+04 |
| 03BMB_H | | 18,8 | 1,99 | 1,51 | 3.5 | 7.048E+06 | 1.139E+07 | | 3.025E+06 | 1,967E+03 | 3.659E+05 |
| 04AMB_H | | 13,4 | 2,01 | 0,72 | 2.32 | 1.377E+07 | 4.533E+07 | | 2.035E+07 | 2,740E+03 | 1.816E+06 |
| 04BMB_H | | 15,6 | 1,76 | 0,63 | 5 | 1.297E+07 | 2.386E+07 | | 7.474E+09 | 5,153E+04 | 2.166E+06 |
| 05AMB_H | | 18,7 | 1,74 | 0,79 | 3.96 | 1.501E+06 | 2.575E+06 | | 9.380E+04 | 3,662E+03 | 1.488E+05 |
| 05BMB_H | | 5,6 | 1,84 | 0,62 | 1.3 | 8.962E+05 | 9.057E+05 | | 5.003E+04 | 1,294E+04 | 7.832E+04 |
| 06AMB_H | | 17,2 | 1,92 | 0,88 | 12.9 | 2.521E+06 | 4.348E+06 | | 5.220E+05 | 5,406E+04 | 2.22E+05 |
| 06BMB_H | | 16,5 | 1,94 | 0,83 | 11.7 | 2.503E+06 | 9.499E+05 | | 1.580E+05 | 1,815E+04 | 3.10E+05 |
| 07AMB_H | | 9,3 | 2,2 | 1,07 | 1.36 | 1.386E+07 | 2.343E+07 | | 1.824E+06 | 2,134E+04 | 3.360E+04 |
| 07BMB_H | | 9,6 | 2,03 | 0,76 | 3.6 | 4.067E+06 | 1.559E+07 | | 1.701E+06 | 5,535E+03 | 4.228E+04 |
| 08AMB_H | | 9,5 | 2,1 | 1,84 | 2.5 | 2.586E+05 | 1.645E+06 | | 2.661E+04 | 6,038E+03 | 1.443E+05 |
| 08BMB_H | | 9,7 | 1,98 | 1,9 | 9.2 | 2.025E+05 | 8.388E+05 | | 2.888E+04 | 2,902E+04 | 1.025E+05 |

**Table S8**. Summary of the results of DNA extraction using Nucleospin® Food Kit, with the modification of bead treatment in the cell lysis step on the quantity and quality of DNA

|  | Results of DNA extraction from sausages | | | | | | | | | | |
| --- | --- | --- | --- | --- | --- | --- | --- | --- | --- | --- | --- |
| Sample ID | | NanoDrop | | | Qubit  ng/μl |  | | Copy Number | | | |
|  |  | ng/μl | 260/280 | 260/230 |  | V3V4 | *Lactobacillus* | | *Bacillus* | *Enterococcus* | ITS |
| 01ANS_B | | 6,6 | 1,44 | 0,53 | 13.3 | 1.880E+06 | 4.856E+06 | | 3.796E+06 | 3,676E+03 | 9.031E+04 |
| 01BNS_B | | 12,9 | 1,74 | 0,79 | 22.2 | 4.097E+06 | 8.461E+06 | | 7.585E+06 | 4,484E+03 | 1.000E+05 |
| 02ANS_B | | 13,3 | 1,57 | 0,56 | 8.52 | 7.969E+06 | 1.404E+07 | | 1.249E+06 | 4,162E+03 | 1.325E+06 |
| 02BNS_B | | 12,5 | 1,49 | 0,54 | 16.6 | 1.239E+06 | 2.326E+06 | | 9.912E+06 | 8,198E+03 | 1.281E+05 |
| 03ANS_B | | 32,9 | 2,04 | 1,34 | 22.8 | 9.950E+06 | 2.381E+07 | | 1.641E+06 | 1,135E+03 | 6.341E+05 |
| 03BNS_B | | 27 | 2,03 | 1,82 | 14.4 | 5.709E+06 | 1.201E+07 | | 3.509E+07 | 1,340E+03 | 1.387E+05 |
| 04ANS_B | | 39,3 | 1,98 | 1,54 | 17.2 | 1.052E+07 | 1.787E+07 | | 4.373E+05 | 2,613E+04 | 3.15E+06 |
| 04BNS_B | | 34 | 2,1 | 1,61 | 8.78 | 2.031E+07 | 4.675E+07 | | 7.632E+05 | 9,874E+03 | 2.23E+06 |
| 05ANS_B | | 15,1 | 2,03 | 1,31 | 17 | 2.697E+06 | 6.158E+06 | | 4.195E+05 | 1,037E+03 | 2.769E+05 |
| 05BNS_B | | 19,1 | 2,02 | 1,25 | 9.28 | 1.892E+06 | 5.291E+06 | | 7.455E+06 | 4,046E+03 | 1.906E+05 |
| 06ANS_B | | 26,3 | 1,77 | 1,48 | 24.2 | 5.635E+06 | 1.195E+07 | | 1.491E+07 | 1,848E+04 | 1.605E+06 |
| 06BNS_B | | 24,5 | 1,74 | 1,45 | 14.5 | 5.157E+06 | 3.094E+07 | | 9.043E+06 | 1,072E+04 | 1.807E+06 |
| 07ANS_B | | 16,3 | 1,79 | 1,31 | 12.3 | 9.002E+06 | 3.044E+07 | | 9.675E+06 | 4,948E+03 | 1.985E+05 |
| 07BNS_B | | 21,2 | 1,69 | 0,99 | 6.78 | 6.274E+06 | 1.185E+07 | | 7.557E+05 | 3,458E+03 | 1.922E+05 |
| 08ANS_B | | 16,9 | 1,51 | 1,01 | 3.4 | 5.740E+05 | 9.527E+05 | | 3.345E+05 | 2,477E+03 | 1.069E+05 |
| 08BNS_B | | 10,5 | 1,62 | 1,16 | 11.4 | 7.764E+05 | 1.461E+06 | | 3.796E+06 | 1,39E+03 | 9.077E+04 |

**Table S9**. Summary of the results of DNA extraction using Nucleospin® Food Kit, with the modification of enzymatic treatment in the cell lysis step on the quantity and quality of DNA

|  | Results of DNA extraction from sausages | | | | | | | | | | |
| --- | --- | --- | --- | --- | --- | --- | --- | --- | --- | --- | --- |
| Sample ID | | NanoDrop | | | Qubit  ng/μl |  | | Copy Number | | |  |
|  |  | ng/μl | 260/280 | 260/230 |  | V3V4 | *Lactobacillus* | | *Bacillus* | *Enterococcus* | ITS |
| 01ANS_E | | 13,9 | 1,55 | 0,72 | 25.6 | 2.815E+06 | 5.265E+06 | | 1.692E+06 | 4,694E+03 | 1.053E+05 |
| 01BNS_E | | 12,5 | 1,65 | 0,74 | 23.1 | 3.009E+06 | 4.187E+06 | | 1.028E+06 | 5,352E+03 | 3.440E+04 |
| 02ANS_E | | 7,4 | 1,42 | 0,65 | 7.24 | 2.412E+06 | 1.800E+06 | | 9.656E+06 | 2,054E+04 | 4.65E+05 |
| 02BNS_E | | 8 | 1,38 | 0,61 | 6.5 | 2.205E+06 | 1.925E+06 | | 8.332E+06 | 1,614E+04 | 2.05E+05 |
| 03ANS_E | | 7,7 | 1,30 | 0,65 | 12.7 | 1.360E+06 | 3.292E+06 | | 9.541E+05 | 2,361E+03 | 4.845E+05 |
| 03BNS_E | | 6,9 | 1,48 | 0,68 | 8.9 | 1.269E+06 | 2.647E+06 | | 1.088E+06 | 1,969E+03 | 4.905E+05 |
| 04ANS_E | | 6,3 | 1,55 | 0,62 | 7.98 | 5.927E+06 | 1.498E+07 | | 4.727E+06 | 4,799E+04 | 3.669E+06 |
| 04BNS_E | | 7 | 1,54 | 0,69 | 6.81 | 7.678E+06 | 1.127E+07 | | 8.838E+06 | 7,085E+04 | 1.019E+07 |
| 05ANS_E | | 8,8 | 1,56 | 0,79 | 12.9 | 5.014E+05 | 7.230E+05 | | 6.940E+04 | 7,994E+02 | 4.981E+05 |
| 05BNS_E | | 8,5 | 1,7 | 0,76 | 11.8 | 4.576E+05 | 5.005E+05 | | 7.183E+04 | 3,774E+03 | 5.373E+05 |
| 06ANS_E | | 16,5 | 1,89 | 1,39 | 42 | 1.905E+05 | 4.416E+05 | | 7.265E+05 | 6,929E+03 | 1.375E+06 |
| 06BNS_E | | 17 | 1,89 | 1,21 | 37.2 | 2.702E+05 | 9.453E+05 | | 7.949E+05 | 1,794E+04 | 3.196E+06 |
| 07ANS_E | | 7,2 | 1,7 | 0,7 | 12.2 | 2.573E+05 | 2.433E+05 | | 9.718E+05 | 1,663E+03 | 5.245E+04 |
| 07BNS_E | | 9,1 | 1,7 | 0,77 | 13.5 | 3.729E+05 | 1.634E+05 | | 1.322E+06 | 1,138E+03 | 2.497E+04 |
| 08ANS_E | | 8,3 | 1,67 | 1,06 | 24.8 | 2.257E+05 | 1.141E+04 | | 2.276E+04 | 2,959E+02 | 7.84E+04 |
| 08BNS_E | | 7,9 | 1,87 | 0,98 | 25.3 | 1.927E+05 | 1.358E+04 | | 1.500E+04 | 4,379E+03 | 1.33E+04 |

**Table S10.** Summary of the results of DNA extraction using Nucleospin® Food Kit, with the modification of thermal treatment in the cell lysis step on the quantity and quality of DNA

|  | Results of DNA extraction from sausages | | | | | | | | | | | |
| --- | --- | --- | --- | --- | --- | --- | --- | --- | --- | --- | --- | --- |
| Sample ID | | NanoDrop | | | Qubit  ng/μl |  | | Copy Number | | | | |
|  |  | ng/μl | 260/280 | 260/230 |  | V3V4 | *Lactobacillus* | | *Bacillus* | *Enterococcus* | | ITS |
| 01ANS_H | | 28,8 | 1,88 | 1,66 | 4.62 | 1.353E+05 | 2.450E+07 | | 6.250E+06 | 8,072E+03 | 8.435E+04 | |
| 01BNS_H | | 34,4 | 1,87 | 1,53 | 15.6 | 8.751E+04 | 2.556E+07 | | 9.062E+06 | 3,614E+03 | 9.053E+04 | |
| 02ANS_H | | 20,7 | 1,7 | 1 | 4.56 | 1.214E+07 | 1.292E+07 | | 8.343E+06 | 1,892E+04 | 3.200E+06 | |
| 02BNS_H | | 16 | 2,01 | 1,32 | 1.46 | 8.006E+06 | 1.279E+07 | | 9.550E+06 | 7,170E+04 | 3.109E+06 | |
| 03ANS_H | | 25,6 | 1,78 | 1,42 | 9.06 | 8.110E+06 | 1.395E+07 | | 1.531E+07 | 3,418E+03 | 3.676E+06 | |
| 03BNS_H | | 24 | 1,75 | 0,72 | 2.84 | 9.638E+06 | 2.513E+03 | | 6.066E+06 | 6,668E+03 | 1.374E+06 | |
| 04ANS_H | | 12,1 | 1,73 | 0,95 | 1.78 | 3.109E+07 | 8.172E+08 | | 3.733E+07 | 1,125E+05 | 4.053E+06 | |
| 04BNS_H | | 18 | 1,86 | 1,07 | 2.14 | 3.937E+07 | 1.090E+10 | | 3.626E+07 | 1,820E+05 | 3.519E+06 | |
| 05ANS_H | | 12,6 | 1,45 | 0,98 | 9.6 | 1.102E+06 | 1.985E+06 | | 1.891E+05 | 2,324E+03 | 3.356E+05 | |
| 05BNS_H | | 6,1 | 1,73 | 1,12 | 2.14 | 1.203E+06 | 9.522E+05 | | 8.286E+04 | 2,938E+03 | 8.371E+04 | |
| 06ANS_H | | 40,4 | 1,89 | 1,58 | 23.2 | 1.620E+06 | 1.155E+06 | | 9.262E+06 | 2,794E+04 | 1.02E+04 | |
| 06BNS_H | | 30,3 | 1,84 | 1,21 | 8.86 | 1.324E+06 | 7.887E+05 | | 5.739E+03 | 4,341E+03 | 2.64E+04 | |
| 07ANS_H | | 18,3 | 1,67 | 0,68 | 3 | 6.645E+06 | 7.184E+06 | | 2.392E+06 | 5,371E+03 | 1.076E+05 | |
| 07BNS_H | | 18,7 | 1,70 | 0,94 | 2.94 | 3.081E+06 | 4.183E+06 | | 2.179E+06 | 5,162E+03 | 9.495E+04 | |
| 08ANS_H | | 16,4 | 1,57 | 0,92 | 4.76 | 3.098E+05 | 9.655E+04 | | 4.120E+04 | 6,275E+02 | 1.254E+05 | |
| 08BNS_H | | 17,6 | 1,7 | 1,16 | 15 | 6.071E+05 | 1.241E+05 | | 1.520E+05 | 5,425E+03 | 4.194E+05 | |

**Table S11**. Selected bacteria calculation of 16S rRNA copy number

| Bacterium | Concentration (ng/μl) | Genome size/weight | | 1 ng gDNA contains | 16S rDNA copies /genome | 1 ng gDNA contains |
| --- | --- | --- | --- | --- | --- | --- |
| *B. lactis* | 0.1 | 2x10^6^ bp | 0.002 pg | 4.562×10^5^ | 4 | 1.825x10^6^ |
| *B. subtilis* | 1 | 4214810 bp | 0.004pg | 5.41x10e^5^ | 10 | 5.41x10e6 |
| *E. faecalis* | 1 | 3026016 bp | 0.003 pg | 3.3x10^5^ | 4 | 1.32x10e^6^ |
| *Lat. sakei* | 10 | 199341 bp | 0.002 pg | 4.711×10^5^ | 5-8 median 7 | 1.65x10e^6^ |

**Table S12**. Information regarding identified number of reads, bacterial alpha diversity indexes and observed OTU per analyzed sample.

| Sample-ID | Filtered reads | Denoized reads | Shannon | Simpson | Chao1 | Observed OTUs |
| --- | --- | --- | --- | --- | --- | --- |
| BP_B_1A | 83637 | 52734 | 5.224233 | 0.963886 | 78 | 77 |
| BP_B_2A | 165029 | 112078 | 4.176149 | 0.915553 | 118 | 88 |
| BP_B_2B | 199615 | 89618 | 3.54017 | 0.881099 | 108 | 74 |
| BP_B_3A | 65402 | 47081 | 4.393841 | 0.922917 | 88 | 75 |
| BP_B_3B | 43863 | 16663 | 3.479265 | 0.885514 | 35 | 34 |
| BP_B_4A | 57268 | 43377 | 3.594215 | 0.880822 | 61 | 60 |
| BP_B_4B | 37296 | 28853 | 3.61376 | 0.885066 | 54 | 51 |
| BP_B_5A | 58135 | 39699 | 3.875437 | 0.880162 | 117 | 109 |
| BP_B_5B | 35379 | 23511 | 3.722531 | 0.871735 | 85 | 81 |
| BP_B_6A | 55402 | 43589 | 3.785431 | 0.908505 | 53 | 45 |
| BP_B_6B | 87823 | 67457 | 3.733713 | 0.904808 | 57 | 52 |
| BP_B_7A | 50739 | 40542 | 3.346462 | 0.876422 | 38 | 37 |
| BP_B_8A | 23959 | 17008 | 3.18769 | 0.843092 | 41 | 40 |
| BP_B_8B | 17809 | 13255 | 3.866778 | 0.895075 | 45 | 45 |
| BP_E_1A | 63217 | 19632 | 4.166267 | 0.906676 | 76 | 76 |
| BP_E_2A | 120008 | 21351 | 5.822622 | 0.969897 | 161 | 157 |
| BP_E_3A | 122146 | 30760 | 4.47363 | 0.930027 | 104 | 94 |
| BP_E_4A | 133705 | 22080 | 5.240898 | 0.962249 | 84 | 80 |
| BP_E_5A | 109292 | 41373 | 2.889306 | 0.784383 | 111 | 98 |
| BP_E_6A | 55461 | 12456 | 4.333143 | 0.898793 | 77 | 77 |
| BP_H_1A | 66632 | 41363 | 5.305602 | 0.961716 | 95 | 91 |
| BP_H_1B | 236024 | 26419 | 6.222583 | 0.978182 | 246 | 229 |
| BP_H_2A | 39832 | 26443 | 4.735188 | 0.94351 | 89 | 84 |
| BP_H_2B | 103267 | 39511 | 3.228163 | 0.811924 | 97 | 81 |
| BP_H_3A | 73288 | 18048 | 3.849169 | 0.888053 | 67 | 65 |
| BP_H_3B | 77219 | 15677 | 4.247093 | 0.921096 | 71 | 69 |
| BP_H_4A | 81493 | 63072 | 3.519785 | 0.859186 | 89 | 80 |
| BP_H_5A | 57413 | 39838 | 4.047769 | 0.907303 | 113 | 97 |
| BP_H_6A | 75493 | 52460 | 4.647279 | 0.934947 | 130 | 121 |
| BP_H_6B | 41000 | 28423 | 4.715924 | 0.937476 | 116 | 110 |
| BP_H_7A | 58633 | 44847 | 3.645252 | 0.89272 | 70 | 65 |
| BP_H_7B | 58442 | 23379 | 5.222976 | 0.94382 | 143 | 141 |
| BP_H_8A | 46607 | 30208 | 4.938978 | 0.924347 | 204 | 190 |
| BP_H_8B | 167495 | 107029 | 3.540086 | 0.864019 | 75 | 61 |
| MB_B_1A | 82775 | 51883 | 5.268091 | 0.964294 | 83 | 79 |
| MB_B_1B | 39658 | 25492 | 5.275134 | 0.967021 | 64 | 64 |
| MB_B_2A | 47053 | 32052 | 3.689023 | 0.892191 | 42 | 41 |
| MB_B_2B | 97476 | 67519 | 3.895533 | 0.905388 | 66 | 58 |
| MB_B_3A | 42031 | 30871 | 4.484501 | 0.941523 | 49 | 48 |
| MB_B_3B | 33420 | 23692 | 4.40651 | 0.93865 | 45 | 44 |
| MB_B_4A | 51042 | 39754 | 3.527464 | 0.872689 | 56 | 51 |
| MB_B_4B | 18335 | 14140 | 3.191085 | 0.849317 | 34 | 34 |
| MB_B_5A | 193796 | 63488 | 3.160936 | 0.808276 | 156 | 118 |
| MB_B_5B | 56026 | 22619 | 2.654419 | 0.775867 | 59 | 53 |
| MB_B_6A | 45875 | 35316 | 3.627453 | 0.890281 | 45 | 44 |
| MB_B_6B | 50185 | 37485 | 3.704905 | 0.894263 | 44 | 41 |
| MB_B_7A | 57678 | 45919 | 3.196147 | 0.858911 | 38 | 36 |
| MB_B_7B | 63590 | 51272 | 3.279998 | 0.871089 | 42 | 39 |
| MB_B_8B | 34903 | 27468 | 3.306094 | 0.863252 | 63 | 60 |
| MB_E_1A | 100215 | 22853 | 4.225306 | 0.916066 | 82 | 77 |
| MB_E_2A | 163781 | 16739 | 4.863376 | 0.944061 | 91 | 90 |
| MB_E_4A | 196099 | 18508 | 4.863303 | 0.935141 | 95 | 95 |
| MB_E_5A | 69645 | 19043 | 3.260752 | 0.813248 | 77 | 75 |
| MB_H_1A | 87043 | 25163 | 4.239669 | 0.917032 | 82 | 76 |
| MB_H_1B | 56921 | 16851 | 4.149207 | 0.913881 | 72 | 71 |
| MB_H_3A | 107649 | 27535 | 3.615194 | 0.891379 | 53 | 49 |
| MB_H_3B | 96067 | 24156 | 3.670464 | 0.892572 | 56 | 50 |
| MB_H_4A | 164945 | 20942 | 4.778726 | 0.937567 | 75 | 72 |
| MB_H_4B | 129827 | 18227 | 4.638021 | 0.928376 | 73 | 73 |
| MB_H_5A | 60411 | 19431 | 3.341471 | 0.821656 | 118 | 109 |
| MB_H_5B | 48338 | 12798 | 3.902015 | 0.866203 | 97 | 95 |
| MB_H_6A | 105011 | 11557 | 4.710316 | 0.916414 | 113 | 113 |
| MB_H_6B | 53444 | 16173 | 5.42069 | 0.954661 | 136 | 136 |
| MB_H_7B | 245811 | 11531 | 4.000432 | 0.885095 | 90 | 90 |
| NS_B_1A | 41127 | 29017 | 4.468093 | 0.929074 | 78 | 75 |
| NS_B_1B | 62100 | 17172 | 4.167425 | 0.905183 | 79 | 77 |
| NS_B_2A | 129761 | 36411 | 4.524187 | 0.916584 | 156 | 132 |
| NS_B_2B | 58244 | 39208 | 4.391303 | 0.927586 | 80 | 77 |
| NS_B_3A | 58885 | 37820 | 4.425927 | 0.938482 | 49 | 48 |
| NS_B_3B | 61652 | 20275 | 3.69155 | 0.895768 | 45 | 44 |
| NS_B_4A | 98443 | 69934 | 3.55086 | 0.869655 | 77 | 69 |
| NS_B_4B | 56764 | 35445 | 4.167554 | 0.875983 | 400 | 285 |
| NS_B_5A | 46208 | 32691 | 3.916899 | 0.916251 | 50 | 48 |
| NS_B_5B | 108078 | 40472 | 3.568979 | 0.827326 | 186 | 153 |
| NS_B_6A | 117888 | 83082 | 4.063091 | 0.912323 | 102 | 92 |
| NS_B_6B | 46668 | 19769 | 6.58079 | 0.969976 | 670 | 624 |
| NS_B_7A | 91013 | 66774 | 3.521746 | 0.877951 | 87 | 68 |
| NS_B_7B | 33899 | 20601 | 4.350814 | 0.913668 | 139 | 134 |
| NS_B_8A | 45884 | 33568 | 3.766014 | 0.878238 | 92 | 87 |
| NS_B_8B | 50898 | 12973 | 3.709177 | 0.883215 | 51 | 48 |
| NS_E_1A | 97396 | 31019 | 4.474191 | 0.926455 | 101 | 94 |
| NS_E_2A | 129381 | 21915 | 5.278 | 0.95673 | 97 | 95 |
| NS_E_3A | 140996 | 32043 | 4.786661 | 0.943135 | 100 | 91 |
| NS_E_5A | 146771 | 34895 | 4.52752 | 0.898393 | 193 | 171 |
| NS_E_6A | 54546 | 14309 | 5.603758 | 0.961753 | 163 | 163 |
| NS_H_1B | 63247 | 47589 | 3.748236 | 0.878768 | 110 | 101 |
| NS_H_2A | 77734 | 50162 | 4.500912 | 0.927954 | 98 | 85 |
| NS_H_2B | 51635 | 28683 | 6.384858 | 0.974115 | 325 | 288 |
| NS_H_3A | 62285 | 38442 | 4.36433 | 0.921398 | 78 | 67 |
| NS_H_3B | 59552 | 38740 | 5.492371 | 0.967746 | 119 | 112 |
| NS_H_4A | 25033 | 18727 | 3.824004 | 0.89609 | 52 | 52 |
| NS_H_4B | 70828 | 52568 | 3.943562 | 0.88801 | 96 | 89 |
| NS_H_5A | 46017 | 26641 | 4.050941 | 0.873351 | 142 | 133 |
| NS_H_5B | 73558 | 24655 | 3.491652 | 0.831069 | 135 | 118 |
| NS_H_6A | 39155 | 25383 | 5.186418 | 0.952165 | 152 | 142 |
| NS_H_6B | 228311 | 58272 | 5.672429 | 0.953185 | 367 | 295 |
| NS_H_7A | 48883 | 36536 | 3.710745 | 0.885369 | 83 | 81 |
| NS_H_8A | 79938 | 22402 | 6.263226 | 0.976396 | 267 | 256 |
| NS_H_8B | 129435 | 83983 | 4.661673 | 0.932932 | 159 | 121 |
